# Rice stripe virus suppresses jasmonic acid-mediated resistance by hijacking brassinosteroid signaling pathway in rice

**DOI:** 10.1371/journal.ppat.1008801

**Published:** 2020-08-31

**Authors:** Jinlong Hu, Jie Huang, Haosen Xu, Yongsheng Wang, Chen Li, Peizheng Wen, Xiaoman You, Xiao Zhang, Gen Pan, Qi Li, Hongliang Zhang, Jun He, Hongming Wu, Ling Jiang, Haiyang Wang, Yuqiang Liu, Jianmin Wan

**Affiliations:** 1 State Key Laboratory of Crop Genetics and Germplasm Enhancement, Nanjing Agricultural University, Nanjing, People's Republic of China; 2 National Key Facility for Crop Gene Resources and Genetic Improvement, Institute of Crop Science, Chinese Academy of Agricultural Sciences, Beijing, People's Republic of China; University of California, Davis Genome Center, UNITED STATES

## Abstract

Rice stripe virus (RSV) is one of the most destructive viral diseases affecting rice production. However, so far, only one RSV resistance gene has been cloned, the molecular mechanisms underlying host-RSV interaction are still poorly understood. Here, we show that increasing levels or signaling of brassinosteroids (BR) and jasmonic acid (JA) can significantly enhance the resistance against RSV. On the contrary, plants impaired in BR or JA signaling are more susceptible to RSV. Moreover, the enhancement of RSV resistance conferred by BR is impaired in *OsMYC2* (a key positive regulator of JA response) knockout plants, suggesting that BR-mediated RSV resistance requires active JA pathway. In addition, we found that RSV infection suppresses the endogenous BR levels to increase the accumulation of OsGSK2, a key negative regulator of BR signaling. OsGSK2 physically interacts with OsMYC2, resulting in the degradation of OsMYC2 by phosphorylation and reduces JA-mediated defense to facilitate virus infection. These findings not only reveal a novel molecular mechanism mediating the crosstalk between BR and JA in response to virus infection and deepen our understanding about the interaction of virus and plants, but also suggest new effective means of breeding RSV resistant crops using genetic engineering.

## Author summary

Brassinosteroids (BR) and jasmonic acid (JA) play critical roles in responding to various stresses. However, the roles of BR and JA, particularly, the crosstalk between these two phytohormones in viral resistance is still very limited. In this work, we found that both BR and JA positively regulate RSV resistance, and JA pathway is necessary for BR-mediated RSV resistance in rice. RSV infection significantly inhibits the BR signaling pathway and increases the accumulation of OsGSK2. OsGSK2 interacts with and phosphorylates OsMYC2, resulting in the degradation of OsMYC2 and suppression of the JA-mediated RSV resistance response to facilitate virus infection. These findings revealed the molecular mechanism of crosstalk between the BR and JA in response to virus infection and deepen our understanding about the mechanism of RSV resistance.

## Introduction

Rice is one of the most important staple foods, but many kinds of biological stresses severely threaten rice yield and quality. Rice stripe virus disease (RSVD), transmitted by small brown planthoppers (SBPH; *Laodelphax striatellus* Fallén), is one of the most destructive viral diseases affecting rice production. It is wide-spread throughout East Asia, especially in China, Japan and Korea. RSV-infected plants display chlorosis, weakness and necrosis in leaves, abnormal growth and even complete loss of grain yield. In addition to rice, RSV also harms other staple crops, such as wheat, barley and maize. Due to the lack of an effective remediation strategy for this disease, RSV management mainly relies on pesticides to control the SBPH. However, over-use of pesticides for SBPH management has heavily polluted the environment. Thus, breeding of RSV-resistant cultivars is considered one of the most cost-effective and environmentally-friendly strategies.

Major efforts have been made to identify RSV resistance genes. So far, five major RSV resistance QTLs (*Stv-bi*, *qSTV11*^*IR24*^, *qSTV11*^*TQ*^, *qSTV11*^*KAS*^ and *qSTV11*^*SG*^) have been identified and fine mapped on chromosome 11[1–5]. However, only one RSV resistance gene, *qSTV11*^*KAS*^ (*STV11*), has been map-based cloned from RSV resistant rice variety. *STV11* encodes a sulfotransferase, catalyzing the conversion of salicylic acid (SA) to sulphonated SA (SSA), leading to increased SA accumulation in the resistant plants to inhibit viral replication [4, 6]. In addition, several studies demonstrated that microRNA pathway should play an essential role in response to RSV infection. For example, argonaute18 confers RSV resistance by sequestering rice microRNA [7], and miR444 regulated the resistance against RSV infection by up-regulation of OsRDR1 expression [8]. Despite the progress, the mechanisms underlying RSV resistance are still poorly understood.

The plant hormone jasmonic acid (JA) is well known as a key regulator of plant defenses against pathogens including bacteria, fungi and viruses [9, 10]. It has been shown that foliar application of JA triggers the systemic resistance against tobacco mosaic virus (TMV), while silencing of JA biosynthetic or signaling genes in tobacco plants increases susceptibility to TMV [11]. Likewise, MeJA-treated plants accumulate less rice ragged stunt virus (RRSV), while blocking JA biosynthesis results in increased RRSV accumulation [12]. Similarly, a rice JA co-receptor mutant *coi1-13* is more susceptible to rice black-streaked dwarf virus (RBSDV) [13]. Nevertheless, the role of JA in RSV resistance has not been reported.

The plant hormone brassinosteroid (BR) is well known for its role in regulating plant growth and development [14, 15], but relatively little is known about its role in plant defense against pathogens. Recently, several studies revealed that BR might also play a pivotal role in plant antiviral responses, but the results drawn from different studies were somewhat discordant. For example, it was reported that exogenous application of BR significantly enhanced the resistance to TMV as well as cucumber mosaic virus (CMV), but TMV resistance is impaired in plants in which *NbBRI1* is silenced [16–18]. In contrast, exogenous application of BR significantly reduced the resistance to RBSDV, and blocking the BR signaling pathway significantly increased the resistance to RBSDV [13]. The discrepancies regarding roles of BR on different viruses remain to be clarified. Moreover, the role of BR in RSV resistance has not yet been reported.

Hormone networks finely build plant defense system in antagonistic or synergistic manners [19, 20]. The crosstalk between JA and BR have been described in several studies. For instance, silencing *BRI1* suppresses herbivory-elicited accumulation of JA, resulting in impaired resistance to the insect herbivore in *Nicotiana attenuate* [21]. BZR1 can positively regulate JA signaling to increase insect resistance in *Arabidopsis* [22]. Similarly, BRs up-regulate the expression of several genes related to the JA pathway and increase the content of JA upon brown planthopper (BPH) infestation in rice [23]. These studies indicate that BRs have a positive role in regulating JA-mediated resistance. However, the precise mechanism whereby BR regulates the JA pathway has not been reported.

In this study, we found that both BR and JA positively regulate RSV resistance. RSV infection significantly inhibits the BR signaling pathway and increases the accumulation of OsGSK2, a key negative regulator of BR signaling. OsGSK2 interacts with and phosphorylates OsMYC2, a master positive regulator of JA signaling, resulting in the degradation of OsMYC2 and suppression of JA-mediated RSV resistance response.

## Results

### BR positively regulates RSV resistance in rice

**Fig 1:**
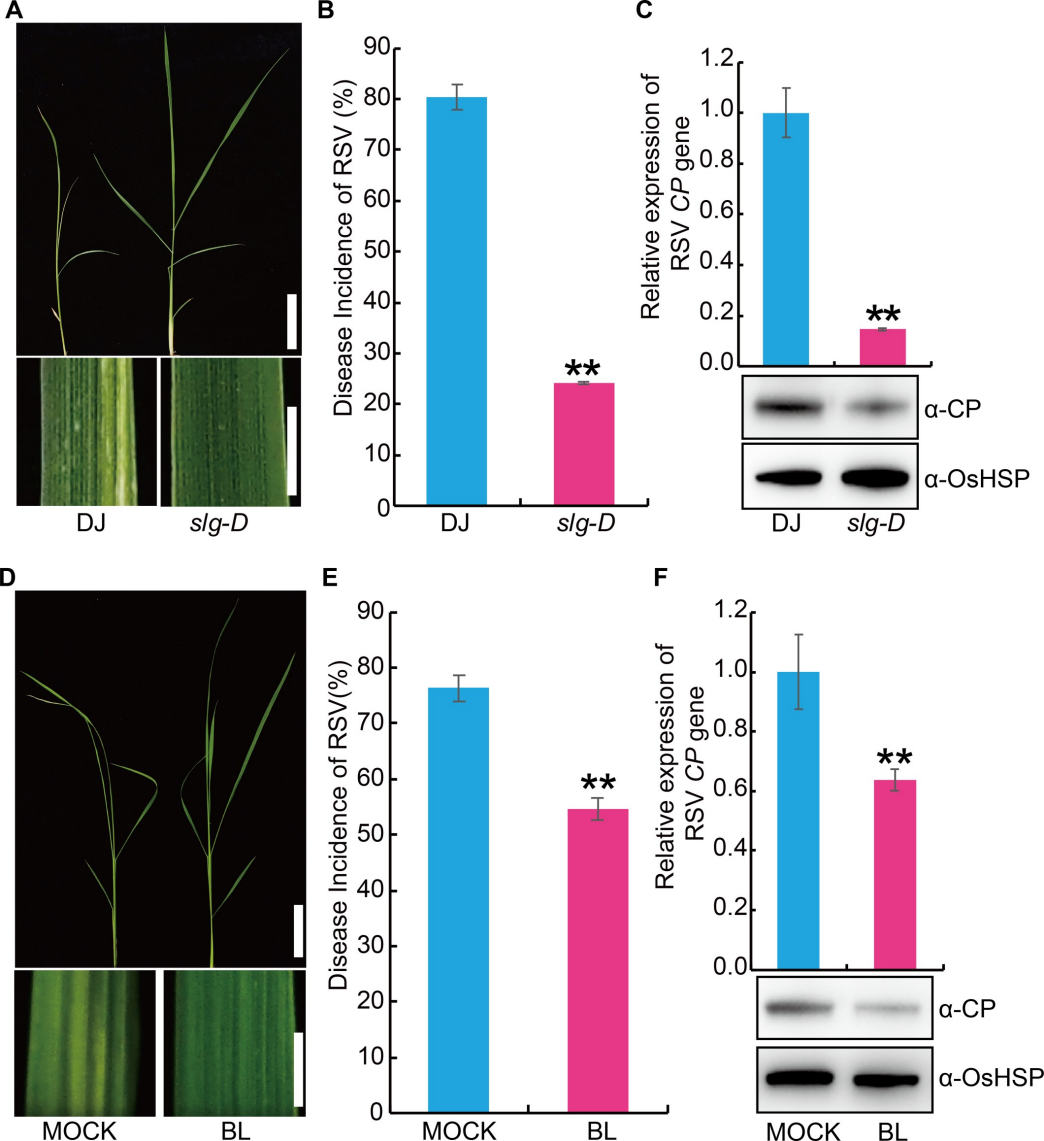
Brassinosteroids (BRs) promote the resistance to rice stripe virus disease (RSV) in rice. (A) Representative images of endogenous BR increased mutant *slender grain Dominant* (*slg-D*) and its wild-type Dongjin (DJ) plants infested with RSV-carrying SBPH. Bar = 5 cm (top). Bar = 3 mm (bottom). (B) RSV incidence of *slg-D* and DJ at 30-day post infection (dpi) with RSV-carrying SBPH. (C) Detection of RSV CP RNA expression levels (upper panel) by quantitative RT–PCR assay and western blot analysis of the RSV coat protein (lower panel) in the RSV-infected plants of *slg-D* and DJ, respectively. OsHSP was used as loading control. (D) Representative images of rice seedling infested with RSV-carrying SBPH after pretreatment with 1 μM epibrassinolide (BL) or MOCK (0.1% Triton X-100). Bar = 5 cm (top). Bar = 3 mm (bottom). (E) RSV incidence of high susceptible rice cultivar Asominori after pretreatment with 1 μM BL or MOCK at 30 dpi with RSV-carrying SBPH. (F) Detection of RSV CP RNA expression levels (upper panel) by quantitative RT–PCR assay and western blot analysis of the RSV coat protein (lower panel) in RSV-infected rice seedling after pretreatment with 1 μM BL or MOCK, respectively. OsHSP was used as loading control. Data are shown as mean ± SEM (n = 3). **P < 0.01 by the Student’s *t*-test in B, C, E and F.

In order to further explore the genetic mechanisms underlying RSV resistance, we screened a collection of activation-tagged T-DNA insertion rice mutants [24, 25]. Among them, one mutant (designated *slender grain Dominant*, *slg-D*), which was reported as a plant with increased levels of endogenous BR [26], displayed higher resistance against RSV than the wild type plant (Fig 1A–1C). This observation suggests that BR could positively regulate RSV resistance. To confirm this, we pre-treated the susceptible rice seedlings with exogenous BR before infection with RSV. The results showed that exogenous BR treatment significantly enhanced RSV resistance of the susceptible rice seedlings compared with the mock treatment (0.1% Triton X-100) (Fig 1D–1F).

**Fig 2:**
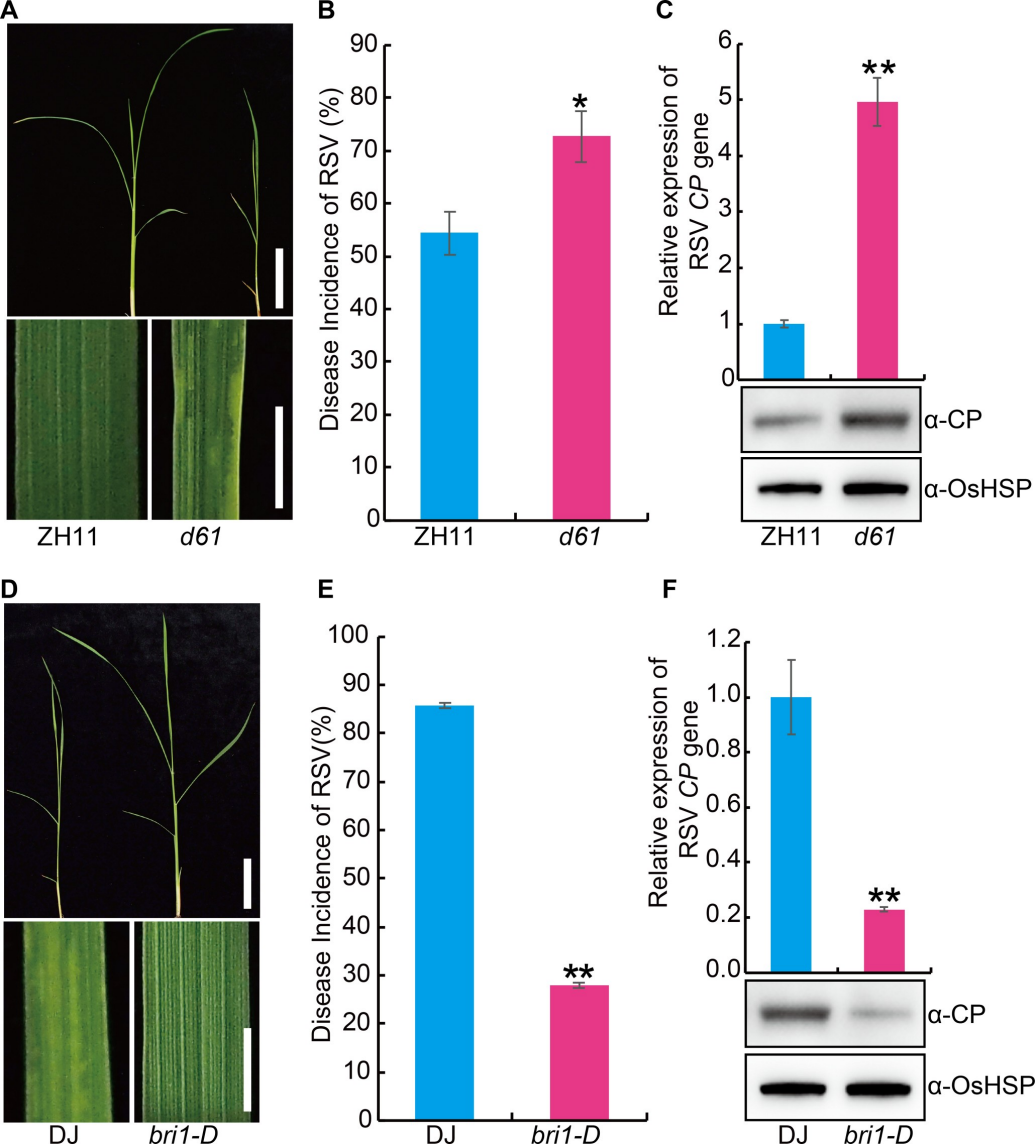
*OsBRI1* plays a positive role in the resistance against RSV in rice. (A) Representative image of the *BR INSENSITIVE 1* (*BRI1*) deficient mutant *d61* and its wild-type Zhonghua11 (ZH11) seedlings infested with RSV-carrying SBPH. Bar = 5 cm (top). Bar = 3 mm (bottom). (B) RSV incidence of *d61* and ZH11 at 30-day post infection (dpi) with RSV-carrying SBPH. (C) Detection of RSV CP RNA expression levels (upper panel) by quantitative RT–PCR assay and western blot analysis of the RSV coat protein (lower panel) in the RSV-infected *d61* and ZH11 plants, respectively. OsHSP was used as loading control. (D) Representative image of the *BRI1-*activated lines *bri1-D* and its wild-type Dongjin (DJ) seedlings infested with RSV-carrying SBPH. Bar = 5 cm (top). Bar = 3 mm (bottom). (E) RSV incidence of *bri1-D* and DJ at 30 dpi with RSV-carrying SBPH. (F) Detection of RSV CP RNA expression levels (upper panel) by quantitative RT–PCR assay and western blot analysis of the RSV coat protein (lower panel) in the RSV-infected *bri1-D* and DJ plants, respectively. OsHSP was used as loading control. Data are shown as mean ± SEM (n = 3). * *P* < 0.05, ** *P* < 0.01 by Student’s *t*-test in B, C, E and F.

**Fig 3:**
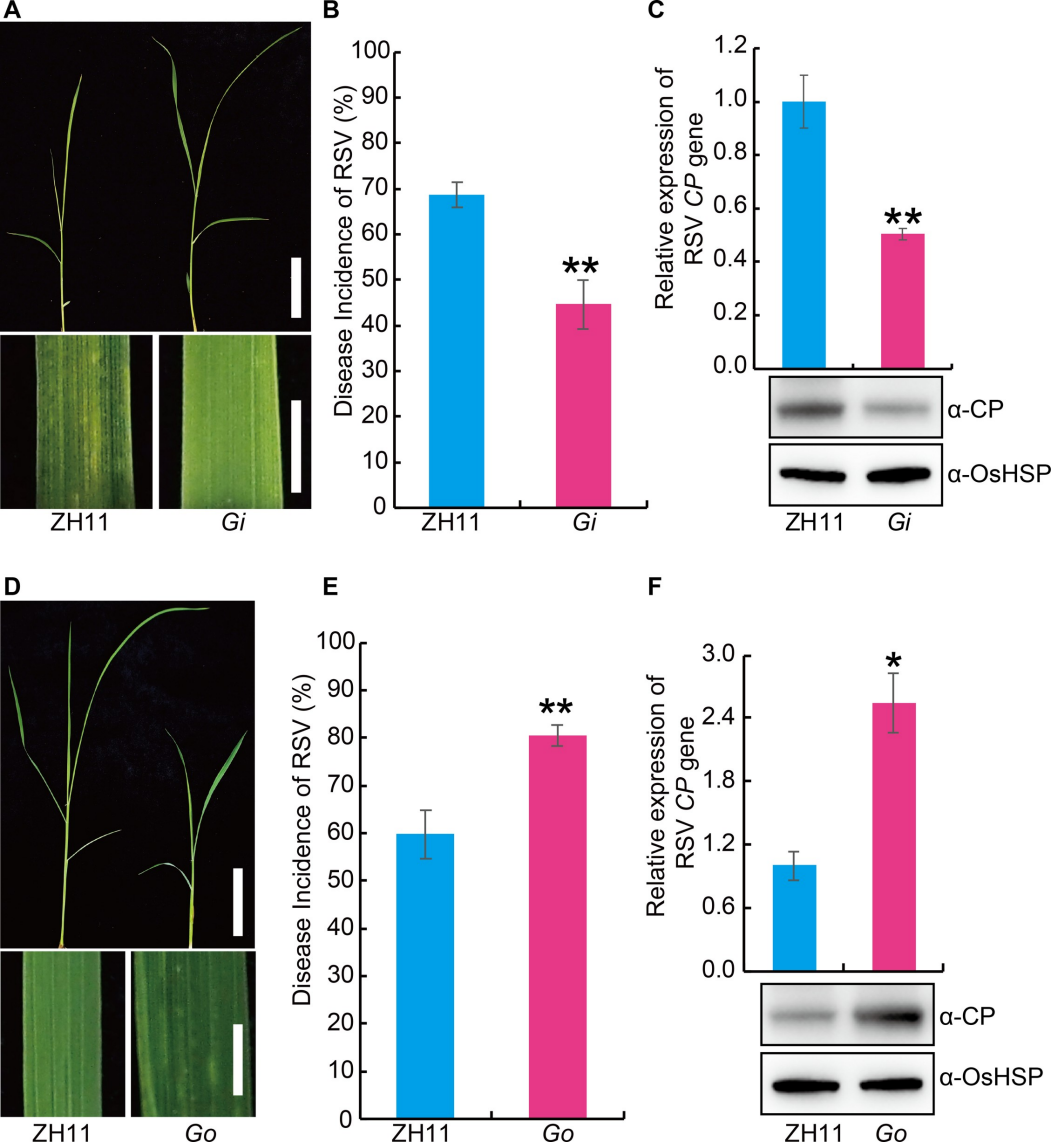
*OsGSK2* negatively regulate the resistance against RSV in rice. (A) Representative images of Zhonghua11 (ZH11) and *GSK2-RNAi* (*Gi*) plants infested with RSV-carrying SBPH. Bar = 5 cm (top). Bar = 3 mm (bottom). (B) RSV incidence of ZH11 and *Gi* at 30 dpi with RSV-carrying SBPH. (C) Detection of RSV CP RNA expression levels (upper panel) by quantitative RT–PCR assay and western blot analysis of the RSV coat protein (lower panel) in the RSV-infected plants of ZH11 and *Gi*, respectively. OsHSP was used as loading control. (D) Representative images of ZH11 and *GSK2* overexpressing (*Go*) plants infested with RSV-carrying SBPH. Bar = 5 cm (top). Bar = 3 mm (bottom). (E) RSV incidence of ZH11 and *Go* at 30 dpi with RSV-carrying SBPH. (F) Detection of RSV CP RNA expression levels (upper panel) by quantitative RT–PCR assay and western blot analysis of the RSV coat protein (lower panel) in the RSV-infected plants of ZH11 and *Go*, respectively. OsHSP was used as loading control. Data are shown as mean ± SEM (n = 3). *P < 0.05, **P < 0.01 by the Student’s *t*-test in B, C, E and F.

To further investigate the role of BR in RSV resistance, we evaluated the RSV resistance of the rice *BR INSENSITIVE 1* (*BRI1*) deficient mutant *d61* [27, 28] and *BRI1-*activated mutant (*bri1-D*) [29]. Compared with the control plants, *d61* displayed greater susceptibility to RSV (Fig 2A–2C), while *bri1-D* was more resistant (Fig 2D–2F). OsGSK2 is a key negative player in BR signaling [30, 31]. We also evaluated the RSV resistance of OsGSK2 knocked-down (*Gi*) or overexpressed (*Go*) plants (S1 Fig). The results showed that the *Gi* plants had significantly enhanced RSV resistance (Fig 3A–3C), while the *Go* plants were more susceptible to RSV than wild type (Fig 3D–3F). To determine whether BR influence the feeding of SBPH, non-preference tests and antibiosis tests were performed. Compared with the wild type plant, mutants or transgenic plants relative to BR pathway displayed no significant difference in the non-preference tests (S2 Fig) and antibiosis tests (S3 Fig). Collectively, these results suggest that BR plays a positive role in RSV resistance.

### JA pathway is necessary for BR-mediated RSV resistance in rice

**Fig 4:**
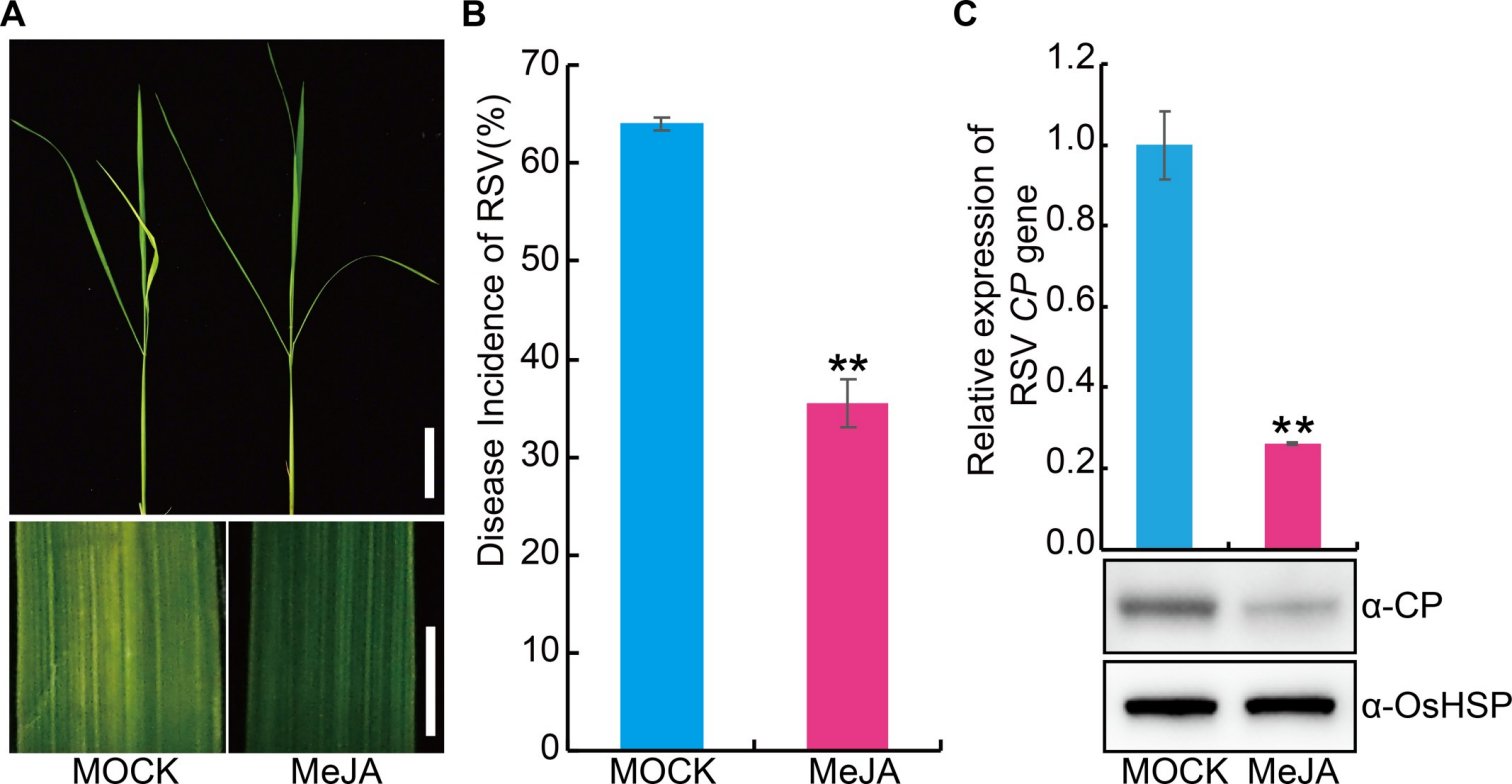
Exogenous application of JA significantly enhances the RSV resistance in rice. (A) Representative image of rice seedlings infested with RSV-carrying SBPH after pretreatment with MOCK (0.1% Triton X-100) or 100 μM MeJA. Bar = 5 cm (top). Bar = 3 mm (bottom). (B) RSV incidence of rice seedlings infested with RSV-carrying SBPH after pretreatment with MOCK or 100 μM MeJA. (C) Detection of RSV CP RNA expression levels (upper panel) by quantitative RT–PCR assay and western blot analysis of the RSV coat protein (lower panel) in the RSV-infected rice seedlings after pretreatment with 100 μM MeJA or MOCK, respectively. OsHSP was used as loading control. Data are shown as mean ± SEM (n = 3). ** *P* < 0.01 by Student’s *t*-test in B and C.

**Fig 5:**
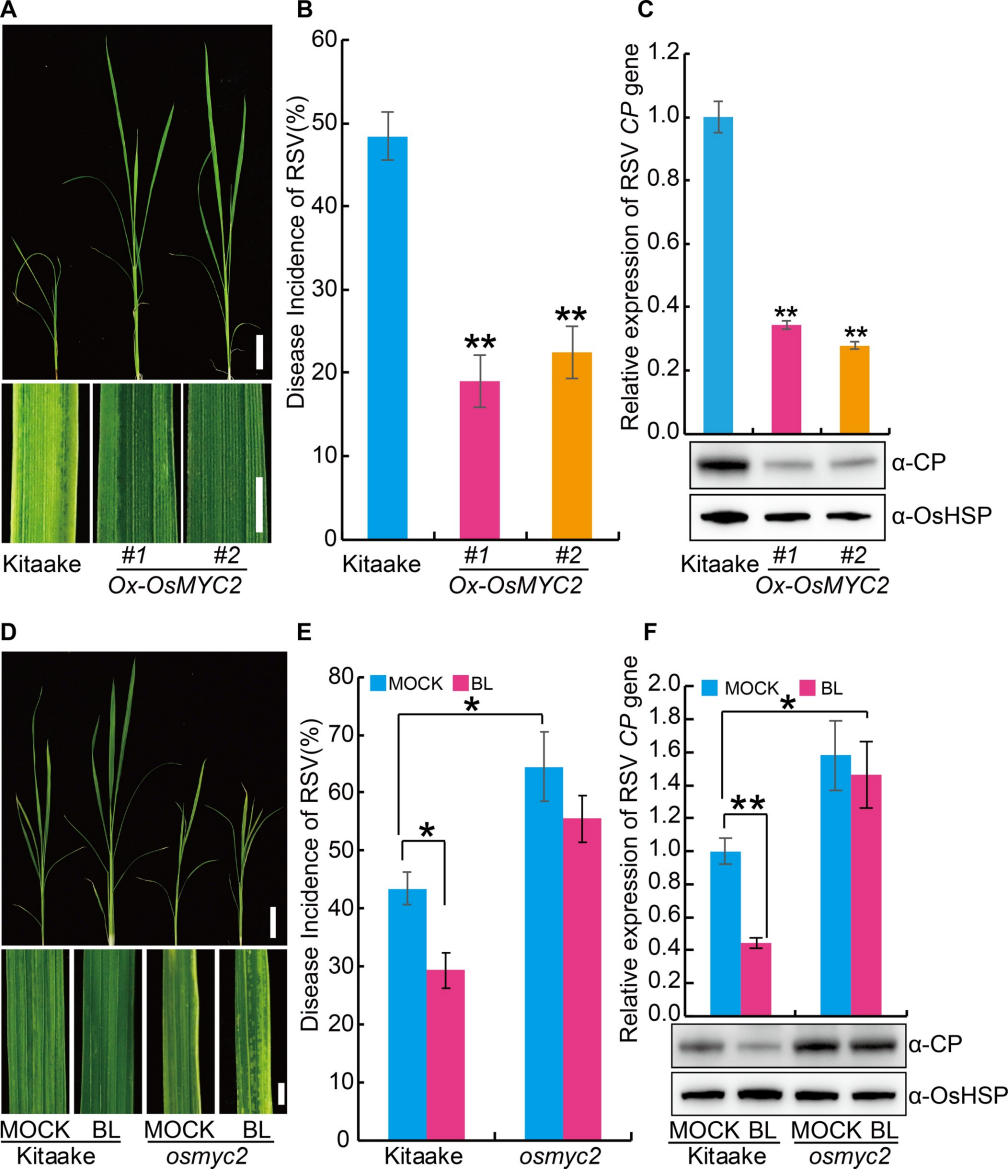
*OsMYC2* plays a positive role in the resistance against RSV in rice. (A) Representative images of Kitaake and *OsMYC2* overexpressing (*Ox-OsMYC2*) plants infested with RSV-carrying SBPH. Bar = 5 cm (top). Bar = 3 mm (bottom). (B) RSV incidence of Kitaake and *Ox-OsMYC2* at 30-day dpi with RSV-carrying SBPH. (C) Detection of RSV CP RNA expression levels (upper panel) by quantitative RT–PCR assay and western blot analysis of the RSV coat protein (lower panel) in the RSV-infected plants of Kitaake and *Ox-OsMYC2*, respectively. OsHSP was used as loading control. (D) Representative images of Kitaake and *OsMYC2* knockout (*osmyc2*) plants infested with RSV-carrying SBPH after pretreatment with 1 μM BL or MOCK (0.1% Triton X-100). Bar = 5 cm (top). Bar = 3 mm (bottom). (E) RSV incidence of Kitaake and *osmyc2* at 30 dpi with RSV-carrying SBPH after pretreatment with 1 μM BL or MOCK. (F) Detection of RSV CP RNA expression levels (upper panel) by quantitative RT–PCR assay and western blot analysis of the RSV coat protein (lower panel) in the RSV-infected plants of Kitaake and *osmyc2* after pretreatment with 1 μM BL or MOCK, respectively. OsHSP was used as loading control. Data are shown as mean ± SEM (n = 3). * *P* < 0.05, ** *P* < 0.01 by the Student’s *t*-test in B, C, E and F.

**Fig 6:**
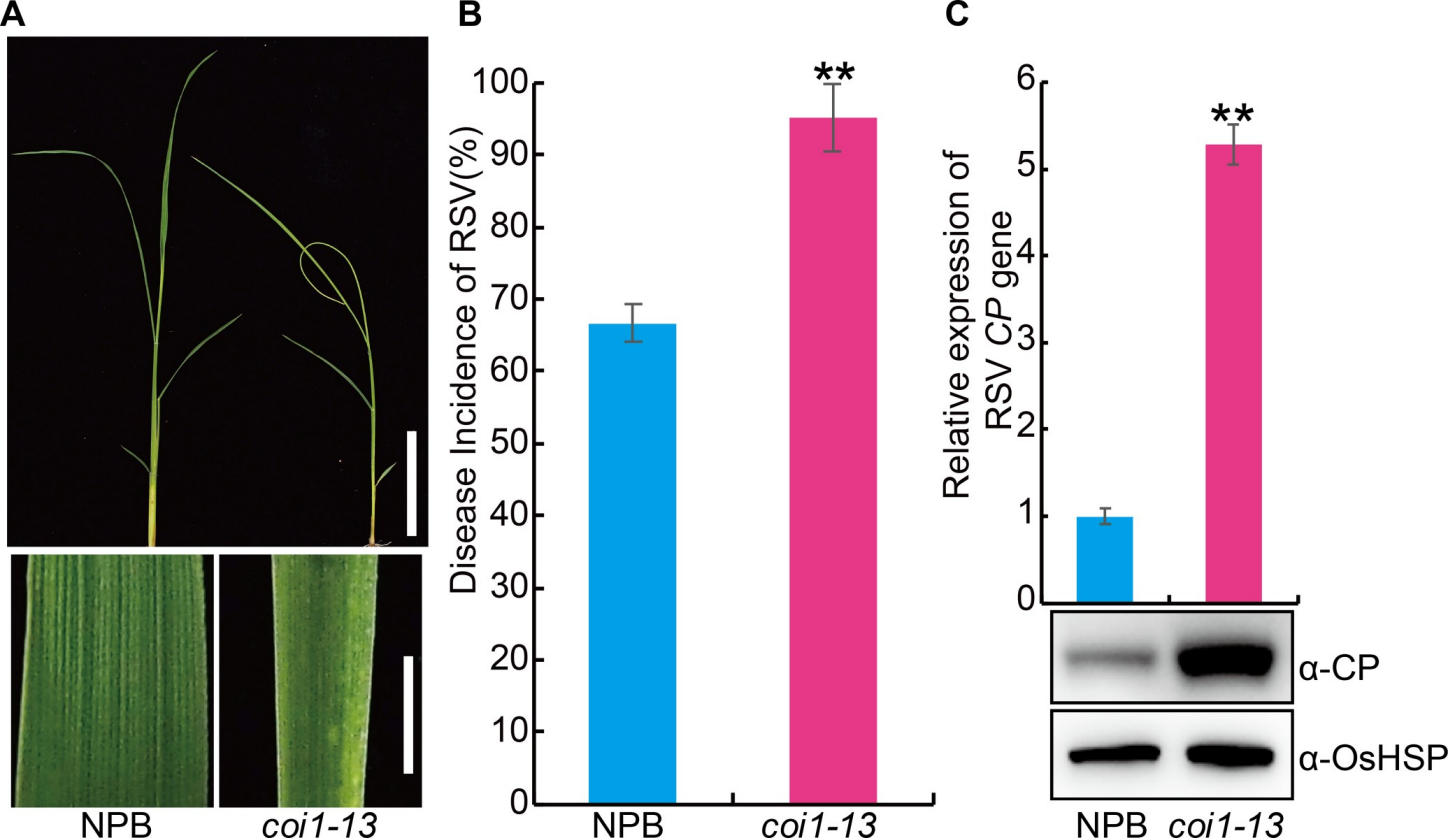
Knockdown of *OsCOI1* expression significantly increases the susceptibility to RSV in rice. (A) Representative image of the JA co-receptor *OsCOI1* RNAi line (*coi1-13*) and the wild-type Nipponbare (NPB) seedlings infested with RSV-carrying SBPH. Bar = 5 cm (top). Bar = 3 mm (bottom). (B) RSV incidence of NPB and *coi1-13* seedlings at 30-day post infection (dpi) with RSV-carrying SBPH. (C) Detection of RSV CP RNA expression levels (upper panel) by quantitative RT–PCR assay and western blot analysis of the RSV coat protein (lower panel) in the RSV-infected NPB and *coi1-13* seedlings, respectively. OsHSP was used as loading control. Data are shown as mean ± SEM (n = 3). ** *P* < 0.01 by Student’s *t*-test in B and C.

In view of the positive role of JA in virus resistance of plants, we first investigated the effect of exogenous JA on RSV resistance. The results showed that exogenous JA application significantly enhanced the RSV resistance (Fig 4A–4C). *OsMYC2* (*LOC_Os10g42430*) is a key positive regulator of the JA pathway in rice [32]. To further explore the role of JA in RSV resistance, we generated transgenic plants overexpressing *OsMYC2* (S4A Fig) and tested its RSV resistance. The results showed plants overexpressing *OsMYC2* displayed higher RSV resistance than the wild-type plants (Fig 5A–5C). Further, we used CRISPR (clustered regularly interspaced short palindromic repeats)/Cas9 technology to knock out *OsMYC2* (S4B Fig) and found that the *osmyc2* knockout lines were more susceptible to RSV than the wild type plants (Fig 5D–5F). Similarly, *coi1-13*, an RNAi line with reduced expression of JA co-receptor *OsCOI1* [33], was also more susceptible to RSV than the wild type plants (Fig 6A–6C). Non-preference tests and antibiosis tests showed that no significant difference in SBPH number (S5A Fig) and SBPH survival rate (S5B Fig) was observed between each JA-related transgenic plants and the wild type plants. These results demonstrate that the JA pathway positively contributes to RSV resistance in rice.

**Fig 7:**
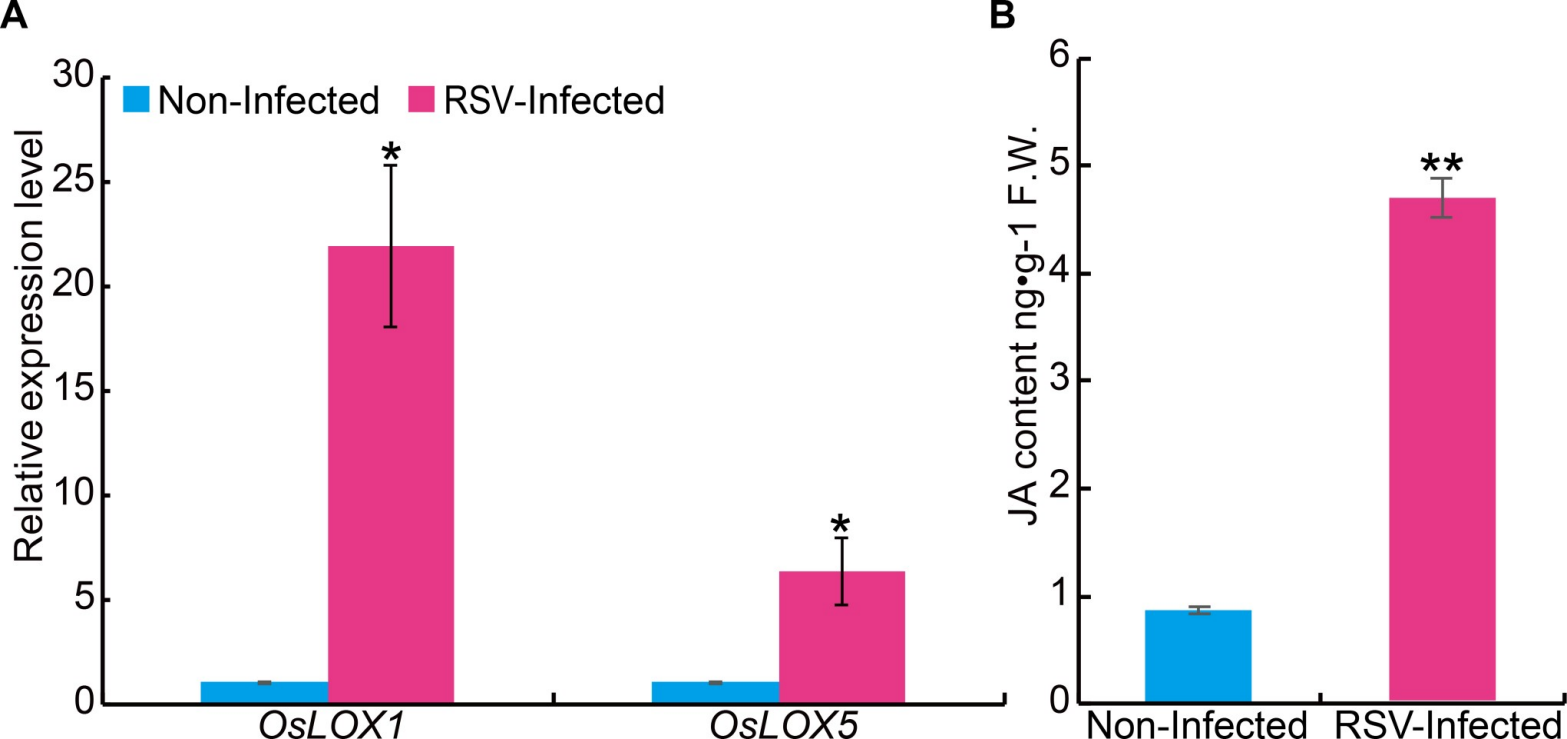
Expression analysis of Jasmonic acid (JA) biosynthetic genes and measurement of JA content in RSV-infected or RSV-free plants. (A) qRT–PCR assay showing the transcript levels of JA biosynthetic genes in RSV-infected and non-infected SYN rice plants. The expression level in RSV free plants was set as 1. (B) Levels of endogenous JA in RSV-infected and non-infected rice seedlings measured by nano-LC–ESI-Q-TOF-MS. Data are shown as mean ± SEM (n = 3). * *P* < 0.05, ** *P* < 0.01 by Student’s *t*-test.

Next, we examined the potential effect of RSV infection on JA biosynthesis. The transcript levels of genes related to JA biosynthesis were determined following RSV infection. The results showed that two genes related to JA biosynthesis (*OsLOX1* and *OsLOX5*) were dramatically upregulated in RSV-infected rice plants in comparison with non-infected plants (Fig 7A). Consistently, the level of endogenous JA content was also significantly increased in RSV-infected plants compared with the non-infected plants (4.67 ng•g^-1^ F.W. vs 0.87 ng•g^-1^ F.W.) (Fig 7B). These results demonstrate that rice plant enhances antiviral defense by increasing the JA level.

To test the relationship between BR and JA in RSV resistance, we evaluated the RSV resistance of the *OsMYC2* knockout plants that were pre-treated with exogenous BL. The results showed that exogenous BL application significantly increased the RSV resistance of wild type plants, but had little effect on the *OsMYC2* knockout plants (Fig 5D–5F). These results suggest that BR-mediated RSV resistance requires active JA signaling pathway.

### OsGSK2 physically interacts with OsMYC2 and regulates its stability

**Fig 8:**
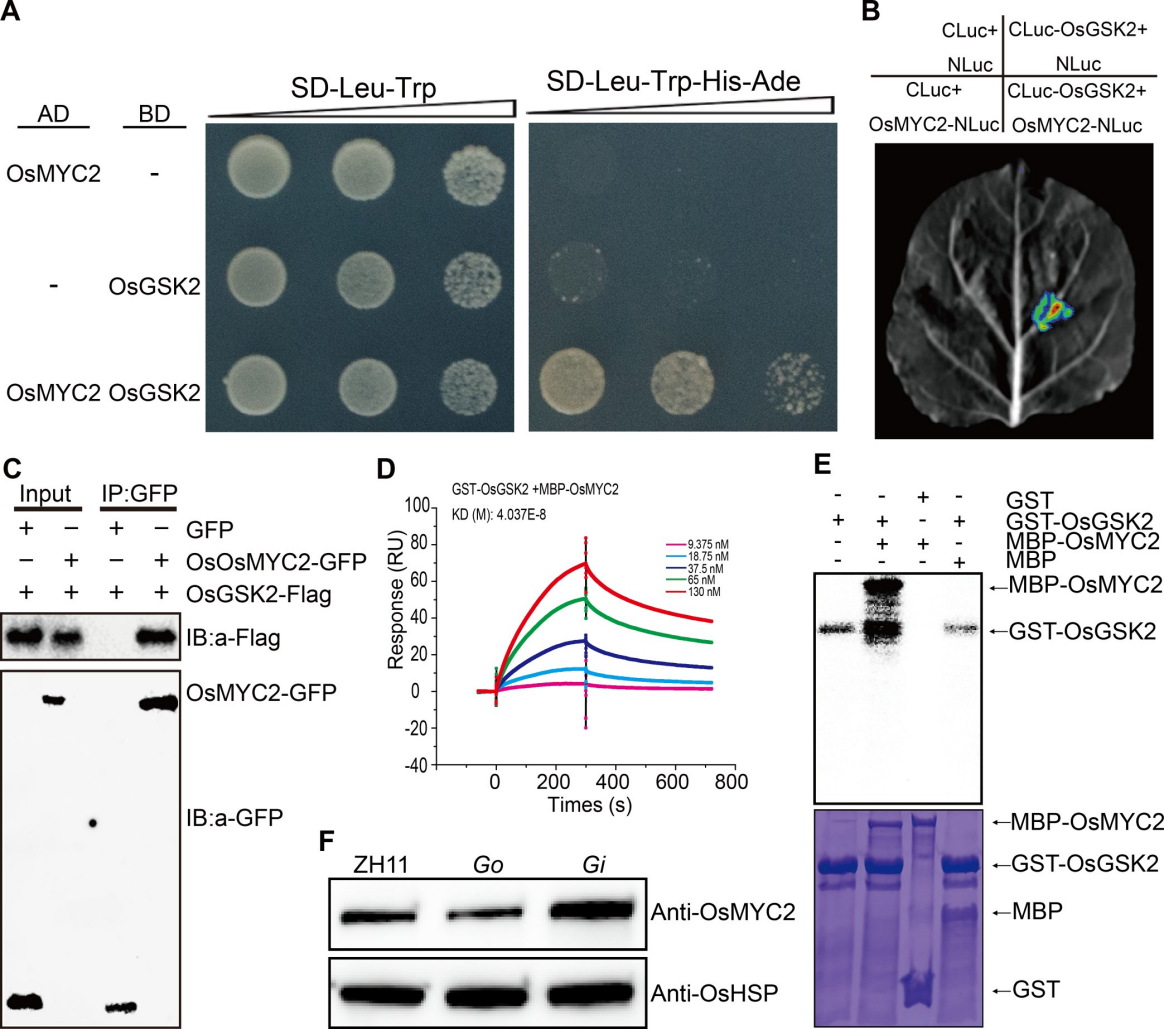
OsGSK2 interacts with and phosphorylates OsMYC2. (A) Interaction between OsGSK2 and OsMYC2 detected in the yeast two-hybrid system. (B) Interaction between OsGSK2 and OsMYC2 detected by Luciferase Complementation Imaging (LCI) assay in *Nicotiana benthamiana*. (C) Interaction between OsGSK2 and OsMYC2 detected by Co-IP assays in *Nicotiana benthamiana*. (D) Interaction between OsGSK2 and OsMYC2 detected by physicochemical analysis. The affinity between GST-OsGSK2 and MBP-OsMYC2 was revealed by dissociation rate constant (KD) (dissociation rate / association rate). (E) *In vitro* phosphorylation assays of GST-OsGSK2 and MBP-OsMYC2. Phosphorylated proteins were detected by autoradiography (top). The proteins used for the phosphorylation assay are shown in the corresponding lower panels with Coomassie blue staining (CBS) (bottom). (F) OsMYC2 protein levels in 14-day seedlings of *Go*, *Gi* and the wild-type Zhonghua11 (ZH11) infested with RSV-carrying SBPH. OsHSP indicates loading control.

To elucidate the molecular mechanisms of BR-mediated antiviral responses, we employed the yeast two-hybrid system using OsGSK2 as bait to find potential interacting proteins. Interestingly, we identified OsMYC2 as an interacting partner of OsGSK2 (Fig 8A). This interaction was further verified by Luciferase Complementation Imaging (LCI) assay (Fig 8B), co-immunoprecipitation (Co-IP) assay (Fig 8C) and *in vitro* Surface Plasmon Resonance (SPR) experiments (Fig 8D, S6A and S6B Fig).

OsGSK2 possesses kinase activity and it usually promotes its substrates for proteasome-mediated degradation by phosphorylation [15, 30, 31, 34]. Thus, we tested whether OsMYC2 is a potential substrate of OsGSK2 using an *in vitro* kinase assay. As expected, OsGSK2 was able to phosphorylate OsMYC2 (Fig 8E). We therefore speculated that OsGSK2 might regulate the stability of OsMYC2. To test this hypothesis, we prepared polyclonal antibodies against OsMYC2 (S7 Fig) and measured the OsMYC2 protein levels in *Go* and *Gi* transgenic plants post infection with RSV. The results showed that OsMYC2 protein levels were obviously increased in the *Gi* transgenic plants, but significantly decreased in the *Go* mutants (Fig 8F). Further, we detected the stability of MBP-OsMYC2 using a cell-free protein degradation system with the total protein extracts from *Go* and *Gi* plants, respectively. Compared with the wild type, the degradation of MBP-OsMYC2 was more rapidly in *Go*, but slowly in *Gi* (S8 Fig). Consistent with these results, we also found that the expression of several genes downstream of *OsMYC2*, including *AK058739* (*subtilisin/chymotrypsin inhibitor*), *AK107891* (*lipid transfer protein*), *AK109913* (*similar to thaumatin-like protein*) and *AK060529* (*beta-1*, *3-glucanase*) [35], were significantly up-regulated in *slg-D*, *bri1-D*, *Gi*, but down-regulated in *d61* and *Go* (S9 Fig). These results demonstrate that OsGSK2 physically interacts with OsMYC2, and promotes its degradation by phosphorylation.

### RSV block JA-mediated RSV resistance by reducing BR biosynthesis

**Fig 9:**
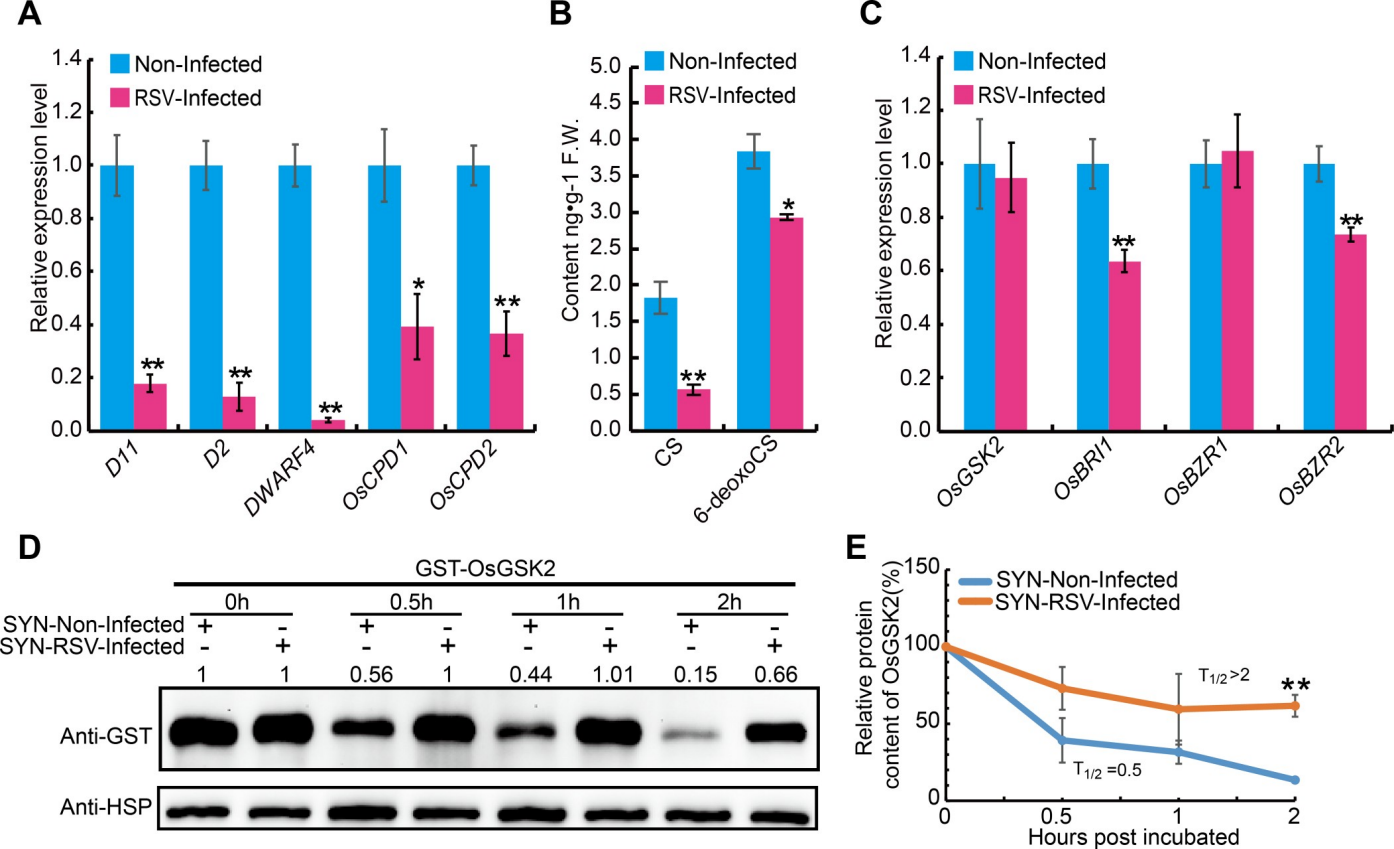
RSV infection suppresses the BR biosynthesis and signal pathway. (A) The relative expression levels of BR biosynthetic genes in non-infected and RSV-infected plants of the susceptible rice cultivar Suyunuo (SYN). (B) Levels of endogenous CS and 6-deoxoCS in RSV-infected and non-infected SYN rice plants. (C) Quantitative RT-PCR analysis showing the relative expression levels of BR signaling-related genes in RSV-infected and non-infected SYN rice plants. (D) Cell-free degradation assay of GST-OsGSK2 incubated with extracts from RSV-infected and non-infected SYN rice plants. OsHSP was used as loading control. The number on the panel indicate the protein level of GST-OsGSK2 relative to its initial value (0 h). The experiment was repeated three times. (E) Half-life plot for cell-free degradation of GST-OsGSK2 in (D). Data are shown as mean ± SEM (n = 3). *P < 0.05, **P < 0.01 by the Student’s *t*-test in A, B, C and E.

In view of the positive role of BR in RSV resistance, transcript levels of several key genes involved in BR biosynthesis and signaling were measured following RSV infection. Quantitative reverse transcription polymerase chain reaction assays (qRT-PCR) showed that genes related to BR biosynthesis, including *D2*, *D11*, *DWARF4*, *OsCPD1* and *OsCPD2*, were significantly down-regulated upon RSV infection (Fig 9A). Consistent with this finding, measurement of BR contents revealed that the concentrations of a major active BR (Castasterone, CS) and the precursor of CS (6-deoxocastasterone, 6-deoxoCS) were both significantly decreased in the RSV-infected plants in comparison with the non-infected plants (Fig 9B).

Moreover, the expression levels of two BR-signaling related genes, *BR INSENSITIVE 1* (*BRI1*) [28] and *BRASSINAZOLE RESISTANT 2* (*OsBZR2*) [36], were also significantly down-regulated in the RSV-infected rice plants (Fig 9C). However, the expression of *OsGSK2* showed no significant change (Fig 9C). As previous studies demonstrated that BR levels could regulate the stability of Arabidopsis BIN2, a close homolog of OsGSK2 [37], we analyzed the influence of RSV infection on the stability of OsGSK2 using a cell-free protein degradation system. The recombinant protein GST-OsGSK2 was incubated with total protein extracts from RSV-infected and uninfected plants, respectively. Compared with the uninfected control, GST-OsGSK2 was more slowly degraded in the extracts prepared from the RSV-infected plants (Fig 9D and 9E). These results suggest that RSV represses BR biosynthesis, resulting in the accumulation of OsGSK2 during the viral infection process.

**Fig 10:**
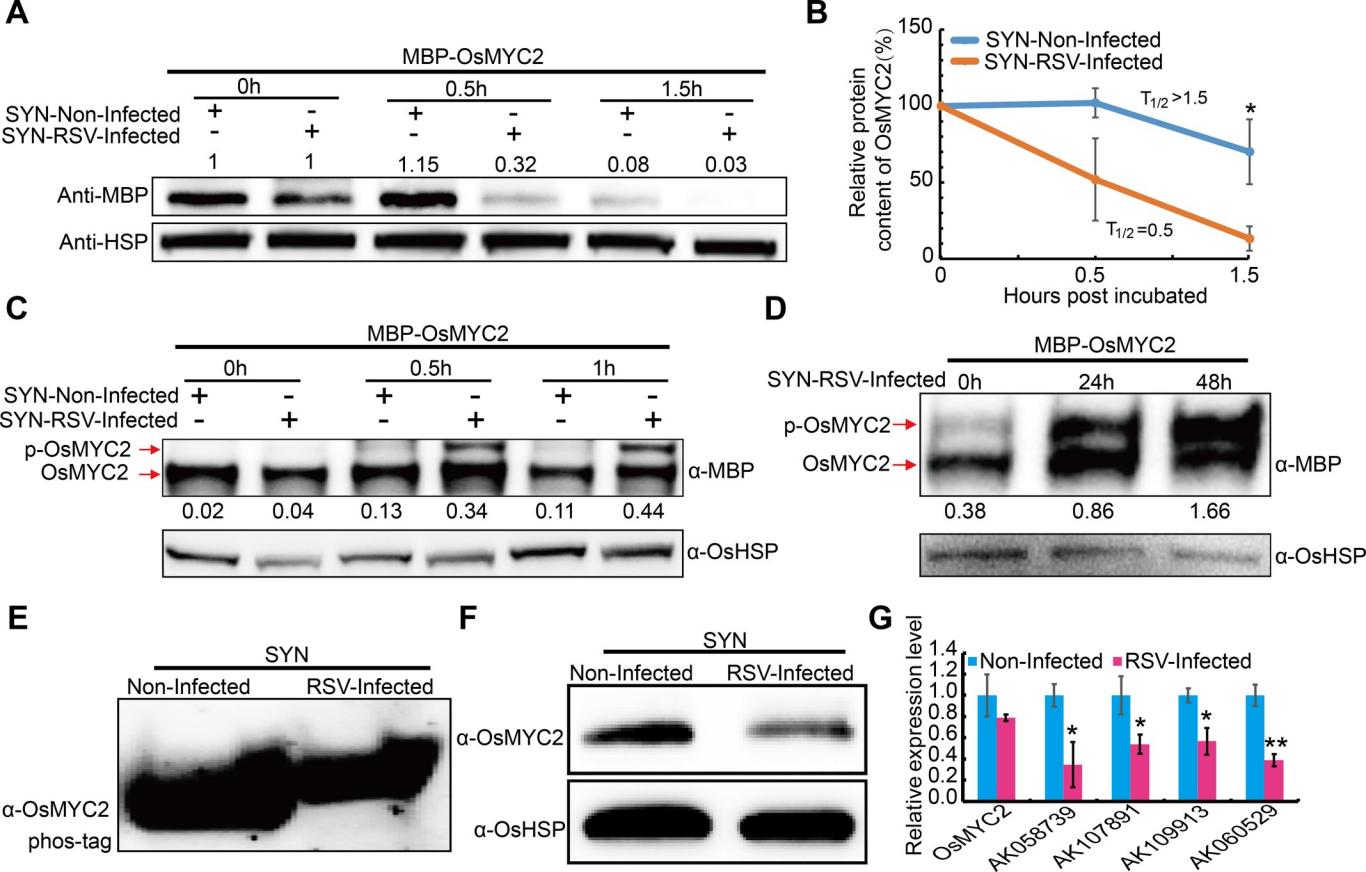
RSV infection induces the degradation of OsMYC2 by phosphorylation. (A) OsMYC2 protein levels in the RSV-infected and non-infected SYN rice plants. OsHSP indicates loading control. The number on the panel indicate the protein level of MBP-OsMYC2 relative to its initial value (0 h). The experiment was repeated four times. (B) Half-life plot for cell-free degradation of MBP-OsMYC2 in (A). (C) Phosphorylation levels of MBP-OsMYC2 incubated with the extracts from RSV-infected and non-infected rice plants using Phos-tag gels. The number under the panel indicate the ratio of pOsMYC2 versus OsMYC2. The experiment was repeated two times. (D) Determination of protein phosphorylation levels incubated with the extracts from rice plants collected at 0, 24 and 48 h post infection with RSV using Phos-tag gels. The number under the panel indicate the ratio of pOsMYC2 versus OsMYC2. The experiment was repeated three times. (E) Phosphorylation analysis of OsMYC2 in RSV-infected and non-infected plants using Phos-tag gels. The experiment was detected four independent RSV-infected and non-infected plants, respectively. (F) OsMYC2 protein levels in the RSV-infected and non-infected SYN rice plants. OsHSP indicates loading control. (G) The transcript levels of representative defense-related genes downstream of *OsMYC2* in RSV-infected and non-infected SYN rice plants. *AK058739* (*subtilisin/chymotrypsin inhibitor*), *AK107891* (*lipid transfer protein*), *AK109913* (*similar to thaumatin-like protein*) and *AK060529* (*beta-1*, *3-glucanase*). Data are shown as mean ± SEM (n > 3). *P < 0.05, **P < 0.01 by the Student’s *t*-test in B and G.

Consistent with the accumulation of OsGSK2 in the RSV-infected plants, we found that MBP-OsMYC2 was rapidly degraded when treated with the extracts of RSV-infected plants (Fig 10A and 10B). Further, MBP-OsMYC2 can be phosphorylated by the extracts prepared from the RSV-infected plants (Fig 10C and 10D), and obvious phosphorylation of OsMYC2 was also detected in the RSV infected plants using OsMYC2 antibody (Fig 10E). Moreover, OsMYC2 protein levels were significantly reduced in RSV infected plants compared to the non-infected plants (Fig 10F). However, this decrease of OsMYC2 protein level in the RSV-infected plants was not paralleled by changes in its transcript levels (Fig 10G). Consistently, the expression levels of the downstream genes of OsMYC2 were also significantly down-regulated after RSV infection (Fig 10G). These results indicate that RSV infection reduces BR biosynthesis and increases OsGSK2, thus leading to reduced accumulation of OsMYC2 and compromised JA pathway to facilitate virus infection.

## Discussion

The regulation of defense signaling networks is complicated and always involves several hormones [38]. The different hormone-signaling pathways enable the plants to precisely regulate the immune response in antagonistic or synergistic manners [39]. On the other hand, in order to overcome plant resistance and benefit their infection, viruses usually disturb phytohormone homeostasis through various means [40]. One way viruses do this is by direct interaction of viral proteins with host proteins to inhibit biosynthesis of plant hormones or reprogram hormonal signaling. For example, the rice dwarf virus outer capsid protein P2 can interact with *ent*-kaurene oxidases and OsIAA10, and then block gibberellin biosynthesis and auxin signaling [41, 42]. In another way, some viruses interfere with hormone pathways by manipulating microRNA. For example, rice ragged stunt virus (RRSV) infection causes increased accumulation of miR319 to decrease endogenous JA levels along with downregulated expression of JA biosynthesis genes to facilitate viral infection and symptom development in rice [12]. Thus, the ultimate defense response of the host plants is usually determined by its interaction with the viruses. Here, we found that RSV infection significantly down-regulated the expression of BR biosynthesis-related genes and decreased endogenous BR levels to suppress the resistance response to RSV. Although a numbers of miRNAs were significantly up-regulated in RSV-infected rice [43, 44], it is not clear whether the microRNA induced by RSV infection can target the BR biosynthesis-related gene to inhibit the biosynthesis of endogenous BR, which needs further study in the future.

BR has been shown to play diverse roles in regulation of viral infections and antiviral responses. Several early studies revealed that BR might play a positive role in plant antiviral responses [16, 18]. However, a recent study showed that BR increases the susceptibility to rice black-streaked dwarf virus (RBSDV) [13]. Here, we showed that both increasing endogenous BR content and exogenous application BR significantly improved the RSV resistance in rice. Meanwhile, enhancing the BR signal by activating *BRI1* or down-regulating *OsGSK2* also confers significantly elevated RSV resistance. In contrast, the *BR INSENSITIVE 1* (*BRI1*) deficient mutant *d61* and *OsGSK2* overexpressing plants were more susceptible to RSV. Hence, we conclude that BR plays a positive role in the defense response against RSV.

We also found that in addition to BR, JA was also involved in RSV resistance. Exogenous JA application or overexpressing *OsMYC2* significantly enhanced RSV resistance, while knockout of *OsMYC2* or knockdown of the JA co-receptor *OsCOI1* significantly reduced the resistance. Furthermore, BR-induced RSV resistance was blocked in the *osmyc2* knockout plants. Collectively, these results demonstrate that BR-mediated enhancement of RSV resistance is dependent on active JA pathway. These findings deepen our understanding of the relationship between BR and JA in defense against RSV infection.

Though previous studies reported crosstalk between BR and JA, their relationship in viral resistance remains unsettled. For example, JA-related genes were significantly induced in plants overproducing BR or exogenously treated with BL during BPH infestation, while they were suppressed in BR-deficient plants, suggesting that BRs positively regulate the JA pathway [23]. However, an antagonistic relationship between BR and JA effects in viral defense has also been reported. For example, BR biosynthetic and signaling genes were downregulated, whereas JA biosynthetic genes were upregulated when rice plants were exposed to RBSDV [13]. It should be noted that the level of downstream signal genes in the JA pathway was not examined in that study. Similarly, we also found that RSV infection significantly downregulated the expression of BR biosynthetic genes and BR levels, but upregulated the expression of JA biosynthetic genes and JA levels. Intriguingly, we found that the expression levels of downstream genes in the JA pathway were significantly reduced after RSV infection. Further, protein interaction experiments demonstrated that OsGSK2 directly interacts with and can phosphorylates OsMYC2, marking it for proteasome-mediated degradation. Together, our results suggest that RSV suppresses JA-mediated resistance by hijacking the BR signaling pathway in rice (S10 Fig). Our studies enrich the knowledge of the crosstalk between plant hormones and virus-host interactions, and provide a valuable strategy for genetically controlling RSV damage in rice production.

## Materials and methods

### Plant materials and growth conditions

Rice mutant or transgenic lines used in this work include *slg-D*, *bri1-D*, *d61*, *coi1-13*, *Go*, and *Gi*. *slender grain Dominant* (*slg-D*) is a dominant mutant in the background of *japonica* cultivar Dongjin (DJ) that overproduces BRs [26]. *bri1-D* is a *BRI1*-activated mutant plant in the background of the *japonica* cultivar Dongjin (DJ) [29]. *d61* is a functionally defective *BRI1* mutant in the background of the *japonica* cultivar Zhonghua11 (ZH11) [30]. *coi1-13* is an *OsCOI1* RNAi transgenic plant in the background of the *japonica* cultivar Nipponbare (NPB) [33]. *Go* and *Gi* are *OsGSK2*-overexpressing and *OsGSK2* RNAi transgenic plants in the background of the *japonica* cultivar Zhonghua11 (ZH11) [30]. Two rice cultivars highly susceptible to RSV, *Oryza sativa* L. *japonica* Suyunuo (SYN) and Asominori (ASO), were used in this study. Rice seedlings were grown in a greenhouse at 25 to 27°C. Rice plants infected with RSV were cultivated in an experimental field at Nanjing under natural long-day conditions.

### Inoculating RSV in rice

SBPHs were maintained on rice seedlings in an insect-rearing room. To obtain viruliferous insects, nymphs were reared on virus-infected rice plants for 2 days, and then maintained on SYN seedlings. About 30 seeds from individual lines or cultivars were sown in a 10 cm-diameter plastic pot with a hole at the bottom. Seedlings were thinned to 25 plants per pot five days after sowing. One-week-old rice plants were inoculated with 5–7 viruliferous SBPH per plant for 48 h. The proportions of healthy plants were calculated 30 days after the inoculation. Plants with yellow and white stripe disease lesions and more severe symptoms were considered susceptible.

### RNA extraction and qRT-PCR

Total RNA of rice samples was extracted with the RNA prep pure Plant kit (TIANGEN Biotech, Beijing, China). About 1 μg total RNA was reversed transcribed with PrimeScript Reverse Transcriptase kit (Takara, Otsu, Japan). Quantitative RT-PCR reactions were performed on an ABI PRISM 7500 device using a SYBR Premix Ex Taq RT–PCR Kit (Takara). Relative transcript levels were calculated by the 2^-ΔΔCT^ method as previously described [45] and the rice Ubiquitin gene (*Os03g0234350*) was used as an internal control. The primers for quantitative RT-PCR analysis are listed in S1 Table.

### Generation of transgenic plants

The *OsMYC2* coding region was amplified with the gene-specific primers 1300-flag-OsMYC2-F/R and cloned into *Xba* I and *Sal* I digested binary vector pCAMBIA1300-221-Flag vector (in which *OsMYC2* is driven by the cauliflower mosaic virus (CaMV) *35S* promoter). To generate the *osmyc2* knockout lines, a 18-bp gene-specific sequence of *OsMYC2* was synthesized and annealed to form the oligo adaptors, and then cloned into the CRISPR-Cas9 expression vector pOs-Cas9 [46, 47]. The recombinant plasmids were transformed into calli of rice Kitaake variety by *Agrobacterium*-mediated method. Hygromycin-resistant calli were grown in artificial incubator to produce transgenic plants. T_0_ plants were grown in padding field of Nanjing Agricultural University and positive plants were confirmed by PCR and sequencing. The positive plant T_2_ seedlings were used for RSV resistance evaluation. Primers for vector construction are listed in S1 Table.

### Hormone treatments

Hormone treatments were performed as described previously [13]. Epibrassinolide (BL, Sigma) and methyl jasmonate (MeJA, Sigma) were dissolved in 100% ethanol to create stock solutions, then diluted with sterile distilled water containing 0.1% Triton X-100 to make the working solutions. 0.1% Triton X-100 containing an equal volume of alcohol was used as a treatment control (MOCK). Rice seedlings of ASO were sprayed with 1 μM BL and 50 μM MeJA, respectively, at 12 h before infection with viruliferous SBPH.

### Evaluation of SBPH resistances

For SBPH antixenosis test, the transgenic or mutant and its wild type seedlings at 1.5–2.0 leaf stage were transferred into the same net cage covered with nylon net and infested with 15 RSV-carrying viruliferous SBPH nymphs per seedling. Then, the number of SBPH on each seedling was counted at 24 h post infestation with SBPH. The observation was recorded for three consecutive days. The average number of insects on each seedling was regarded as the value of antixenosis.

For SBPH antibiosis test, the transgenic or mutant plant and its wild type seedling was transferred into a net cage covered with nylon net at 1.5–2.0 leaf stage, respectively, then each seedling was infested with 25 RSV-carrying viruliferous SBPH nymphs. The number of SBPH was counted at 1, 3, 5 and 7 days post infestation. The average survival rate of SBPH on each seedling was regarded as the value of antibiosis. Details of the method procedures have been described previously [6, 48].

### Quantification of Endogenous JA and BR levels

The leaves of SYN were sampled at 70 days after infection with RSV and then preserved in a -80°C freezer. The endogenous BR and JA contents were determined by the Wuhan Greensword Creation Technology Co. Ltd. BR contents were analyzed using high-performance liquid chromatography electrospray ionisation–tandom mass spectrometry (HPLC–ESI–MS/MS) as described previously [49] with minor modification. Briefly, about 3 g (fresh weight, FW) of flag leaves from RSV-infected or uninfected rice plants were ground into fine powder with liquid nitrogen, and then BRs were extracted with acetonitrile (5 mL/g) overnight at -20°C. Deuterium-labeled BRs [^2^H_3_] BL (4 ng) and [^2^H_3_] CS (4 ng) were added as internal standards for quantification. After centrifugation (10000 rpm, -4–0°C, 10 min), the supernatant was dehydrated and passed through a desorpt C_18_ SPE cartridge. After desorption the solution was evaporated with ethyl ether, and then dissolved in methanol: water (4:1, v/v, 100 μL). 30 μL of the purified product was injected into the HPLC–ESI–MS/MS system for analysis.

JA contents were analyzed using nano-LC–ESI-Q-TOF-MS analysis as described previously [50] with minor modification. Approximately 3 g (FW) of flag leaves from RSV infected or non-infected rice plants were harvested for JA measurement.

### RSV CP and OsMYC2 protein levels analysis

Samples were ground into fine powder in liquid nitrogen, and then suspended in 2 × volumes of protein extraction buffer (100 mM Tris–HCl pH 8.0, 10 mM MgCl2, 18% sucrose, 40mM *β*-mercaptoethanol and 1 × Protease inhibitor cocktail) and incubated at 4°C for 30 min with rotation. After centrifuging at 12,000 g and 4°C for 10 min, the supernatant was boiled in 5 x SDS sample buffer (250mM Tris-HCl, PH = 6.8, 10% SDS, 0.5% bromophenol blue, 50% glycerin, 5% *β*-mercaptoethanol). Protein samples were separated on 10% SDS-PAGE gel and then transferred to the NC membrane, detected with antibodies against RSV CP and OsMYC2, respectively. The monoclonal antibody of RSV CP were friendly provided by Tong Zhou Professor (Institute of Plant Protection, Jiangsu Academy of Agricultural Sciences). OsMYC2 polyclonal antibodies were prepared by ABclonal Biotechnology Co., Ltd, China. OsHSP protein levels detected with antibodies (Beijing Protein Innovation, AbM51099-31-PU) were used as loading control.

### Cell-free degradation

Rice seedlings were harvested and ground into fine powder in liquid nitrogen. Total proteins were subsequently extracted with degradation buffer containing 25 mM Tris-HCl, pH 7.5, 10 mM NaCl, 10 mM MgCl_2_, 5 mM DTT, as previously described [51]. The extract was centrifuged at 12000 rpm for 10 min. The supernatant was collected and adjusted to equal concentration with the degradation buffer. For GST-OsGSK2 and MBP-OsMYC2 protein degradation, about 10 μg of purified proteins were separately mixed with 40 μl rice total-protein extract at 28°C. The reactions were incubated at 28°C for 0, 0.5, 1 and 2 h, then 5 x SDS sample buffer (250mM Tris-HCl, PH = 6.8, 10% SDS, 0.5% bromophenol blue, 50% glycerin, 5% *β*-mercaptoethanol) was added to stop the reactions. Proteins were detected by western blots with HRP-conjugated Anti-GST or Anti-MBP monoclonal antibody at 1:5, 000 dilution, respectively.

### Phosphorylation assay of MBP-MYC2 by the Phos-tag gel

Samples were ground into fine powder in liquid nitrogen, and then suspended in 2 × volumes of protein extraction buffer (100 mM Tris–HCl pH 8.0, 10 mM MgCl2, 18% sucrose, 40mM *β*-mercaptoethanol and 1 × Protease inhibitor cocktail) and incubated at 4°C for 30 min with rotation. The extract was centrifuged at 12000 rpm for 10 min. The supernatant was collected and adjusted to equal concentration with the protein extraction buffer. About 10 μg of MBP-MYC2 were mixed with 20 μl total-protein extract, 1× Protease inhibitor cocktail and 1× MG132 at 37°C, then added 1× phosphatase inhibitor at the end and boiled in 5 x SDS sample buffer. Protein samples were separated on 7.5% Phos-tag SDS-PAGE gels and then transferred to the NC membrane, detected with antibodies against MBP.

### Yeast two-hybrid assay

The full-length CDS of *OsGSK2* was cloned into pGBKT7 as the bait. A yeast two-hybrid library was constructed from the mRNA of rice seedlings. Yeast transformation and screening procedures were performed according to the Clontech Yeast Protocols Handbook (www.clontech.com). Primers used to make the plasmid constructs are listed in S1 Table.

### Luciferase complementation imaging assay

The Luciferase Complementation Imaging (LCI) assay was performed as previously described [52]. The cDNAs of *OsMYC2* and *OsGSK2* were inserted into pCAMBIA 1300-NLuc and pCAMBIA 1300-CLuc to construct the *OsMYC2*-NLuc, CLuc-*OsGSK2* vectors, respectively. These binary vectors were transformed into *Agrobacterium tumefaciens* (strain *EHA105*). Bacterial suspensions were infiltrated into 6-week-old *N*. *benthamiana* leaves using needleless syringes. One millimolar luciferin (Promega) was sprayed onto leaves, and then the leaves were kept in the dark for 6 min to quench the fluorescence. The LUC image was observed with a low-light-cooled CCD imaging apparatus (NightShade LB 985; Berthold).

### Co-IP

Full-length cDNAs of *OsMYC2* and *OsGSK2* were amplified and cloned into the pCAMBIA1305-GFP and pCAMBIA1300-221-Flag vectors, respectively (S1 Table). The *OsMYC2-GFP* and *OsGSK2-Flag* recombinant plasmids were transiently expressed in *Nicotiana benthamiana* leaves by *Agrobacterium* infiltration. Three days after the transfection, total proteins were extracted from freshly-harvested leaves with the NB1 buffer (50 mM Tris-MES, PH 8.0, 1 Mm MgCl2, 0.5 M sucrose, 10 mM EDTA, 5 mM DTT, protease inhibitor cocktail Complete Mini tablets [Roche, 04693132001]) at the mass volume ratio of 1:2. The mixtures were gently shaken for 30 min at 4°C and then the supernatant was collected after centrifugation at 12, 000g, 4°C for 30 min. The IP complexes were pre-cleared with protein G-Agarose (Roche, 11243233001) for 1 h and then were captured with GFP magnetic beads (MBL, D153-11) at 4°C for at least 4 h. The magnetic beads were rinsed three times with the NB1 buffer and then boiled in 2 × SDS sample buffer and were detected by western blots with Anti-GFP (MBL, 598–7) or anti-Flag (MBL, M185-7) antibodies at 1:2, 000 dilutions.

### Affinity and kinetic studies

The interaction between OsGSK2 and OsMYC2 was assayed by a method based on surface plasmon resonance (SPR) using a biosensor instrument (Biacore T200; GE Healthcare). OsMYC2 tagged with maltose-binding protein (MBP) and MBP were initially immobilized on the CM5 sensor chip (GE Healthcare) using the amino-group with a continuous flow of 10 mL per min. Association and dissociation profiles were obtained with a continuous flow of 30 mL per min, using glutathione S-transferase (GST)-tagged OsGSK2 and GST proteins as the analytes at concentrations ranging from 9.375 to 130 nmol/mL and 12.5 to 200 nmol/mL. Kinetic data were obtained using Biacore T200 evaluation software.

### *In vitro* kinase assays

*In vitro* kinase assays were performed in a reaction mixture containing 40 mM Hepes, pH 7.5, 20 mM MgCl_2_, 2 mM DTT, 10 μCi [^32^P] γATP, 1× proteinase inhibitor cocktail, 1× phosphatase inhibitor cocktail, 4 μg MBP-MYC2 and 15 μg GST-OsGSK2 or GST. The mixture was incubated at 30°C for 0.5 h, then 7μl 5× SDS loading buffer were added to terminate the reaction. Thereafter, the reaction products were boiled at 100°C for 5 min, and then separated by 10% SDS-PAGE. The gel was stained with Coomassie blue and imaged, then exposed to GE Amersham hyperfilm MP film for 2 h to detect phosphorylation.

### Statistical analysis

Differences were analyzed using a Student’s *t*-test. All analyses were performed using Microsoft office 2016 excel software. A *P*-value < 0.05 was considered as statistically significant.

## Supporting information

S1 Fig The transcript levels of *OsGSK2* in the *OsGSK2* overexpressing (*Go*), *OsGSK2-RNAi* (*Gi*) and the wild-type Zhonghua11 (ZH11) plants. The expression level of *OsGSK2* in ZH11 was set as 1. All data are shown as mean ± SEM (n = 3). * *P* <0.05 in comparison with the WT plant (Student’s *t*-test).
(TIF)

S2 Fig Feeding preference of SBPH on BR-related mutants. Numbers of SBPH on *slg-D*, Dongjin (DJ), *Gi*, Zhonghua11 (ZH11), *Go*, *bri1-D* and *d61* plants were recorded at 24 h post infestation with SBPH. “ns” indicate no significant difference in comparison with the WT plant (Student’s *t*-test).
(TIF)

S3 Fig Survival rates of SBPH on BR-related mutant. Survival rates of SBPH on *slg-D*, Dongjin (DJ), *Gi*, Zhonghua11 (ZH11), *Go*, *bri1-D* and *d61* plant were recorded on 1, 3, 5 and 7-day post infestation with SBPH. “ns” indicate no significant difference in comparison with the WT plant (Student’s *t*-test).
(TIF)

S4 Fig Expression analysis of *OsMYC2* and the schematic diagram of sgRNA targeting *OsMYC2*. (A) qRT-PCR analysis of the transcript levels of *OsMYC2* in Kitaake, *OsMYC2* over-expressing (*Ox-OsMYC2*) and knockout (*osmyc2*) plants. The expression level of *OsMYC2* in Kitaake was set as 1. *Ubiquitin* (*Os03g0234350*) was used as internal reference. Data are shown as mean ± SEM (n = 3). * *P* < 0.05 by Student’s *t*-test. (B) Diagram of the CRISPR/Cas9 target fragment (in yellow) in the CDS of *OsMYC2*. The position of the PAM sequence is underlined (in red). The minus signs indicate the base deleted in *osmyc2* in comparison with Kitaake.
(TIF)

S5 Fig Feeding preferences and survival rate of SBPH on JA-related mutants. (A) Numbers of SBPH on Nipponbare (NPB), JA co-receptor *OsCOI1* RNAi (*coi1-13*), Kitaake, *OsMYC2* knock out (*osmyc2*) and *OsMYC2* overexpressing (*Ox-OsMYC2*) plants were recorded at 24 h post infestation with SBPH. Data are shown as mean ± SEM (n = 9). (B) Survival rate of SBPH on *coi1-13*, NPB, *osmyc2* and *Ox-OsMYC2* plants on 1, 3, 5 and 7-d post infestation with SBPH. Data are shown as mean ± SEM (n = 5). “ns” indicate no significant difference in comparison with the WT plant (Student’s *t*-test).
(TIF)

S6 Fig Binding analysis of GST with OsMYC2 (A) or OsGSK2 with MBP (B) by physicochemical analysis. The results show that GST cannot bind OsMYC2 and OsGSK2. (TIF)

S7 Fig Western blot analysis of OsMYC2 protein levels in Kitaake, *OsMYC2* overexpressing (*Ox-OsMYC2*) and *OsMYC2* knockout (*osmyc2*) plants using anti-OsMYC2 antibodies. OsHSP was used as an internal reference.
(TIF)

S8 Fig Cell-free degradation assay of MBP-OsMYC2 incubated with extracts from Zhonghua11 (ZH11), *Go* and *Gi* plants. OsHSP was used as loading control. The number on the each panel indicate the protein level of MBP-OsMYC2 relative to its initial value (0 min).
(TIF)

S9 Fig The transcript levels of several *OsMYC2* downstream genes in the *slg-D*, *bri1-D*, *d61*, *Gi* and *Go* plants. All data are shown as mean ± SEM (n = 3). *P < 0.05 by Student’s *t*-test. *AK058739* (*subtilisin/chymotrypsin inhibitor*), *AK107891* (*lipid transfer protein*), *AK109913* (*similar to thaumatin-like protein*) and *AK060529* (*beta-1*, *3-glucanase*).
(TIF)

S10 Fig A proposed model of BR-JA cross-talk mediating RSV resistance in rice. When rice plant is infected with RSV, the biosynthesis of JA is induced, which activates the JA-mediated RSV resistance response. Meanwhile, RSV infection increases the accumulation of OsGSK2 by reducing the level of endogenous BR. OsGSK2 interacts with and phosphorylates OsMYC2, which results in the degradation of OsMYC2 and blocking of the JA signal pathway to benefit viral infection.
(TIF)

S1 Table Primers used in the study. (DOCX)
