# Supplementary figures and images for: Rice stripe virus suppresses jasmonic acid-mediated resistance by hijacking brassinosteroid signaling pathway in rice
Source: PLoS Pathog. 2020 Aug 31;16(8):e1008801. doi: 10.1371/journal.ppat.1008801 (PMC7485985; doi:10.1371/journal.ppat.1008801)

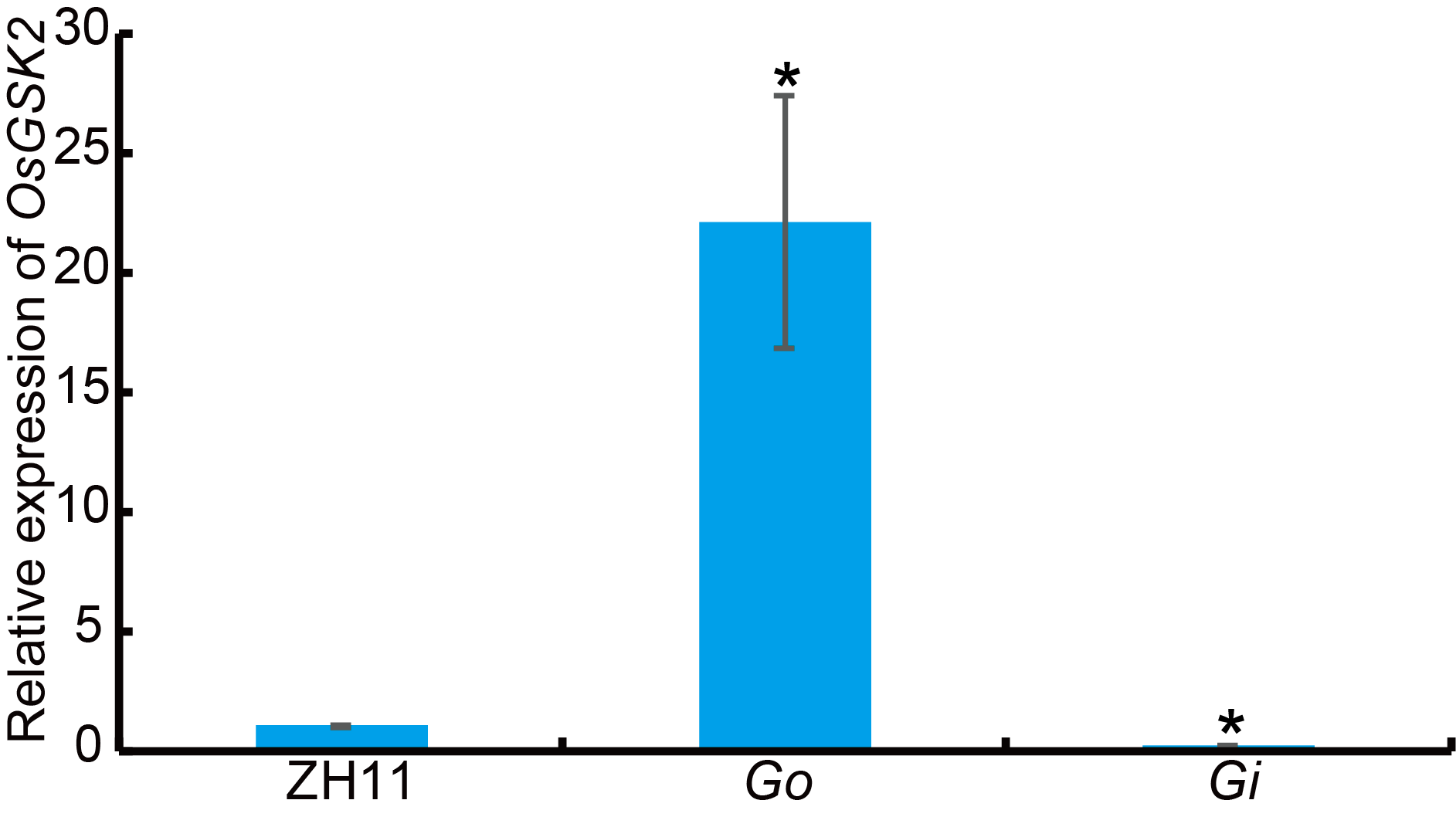

Supplement: S1 Fig — The expression level of OsGSK2 in ZH11 was set as 1. All data are shown as mean ± SEM (n = 3). * P <0.05 in comparison with the WT plant (Student’s t-test). (TIF) [file ppat.1008801.s001.tif]

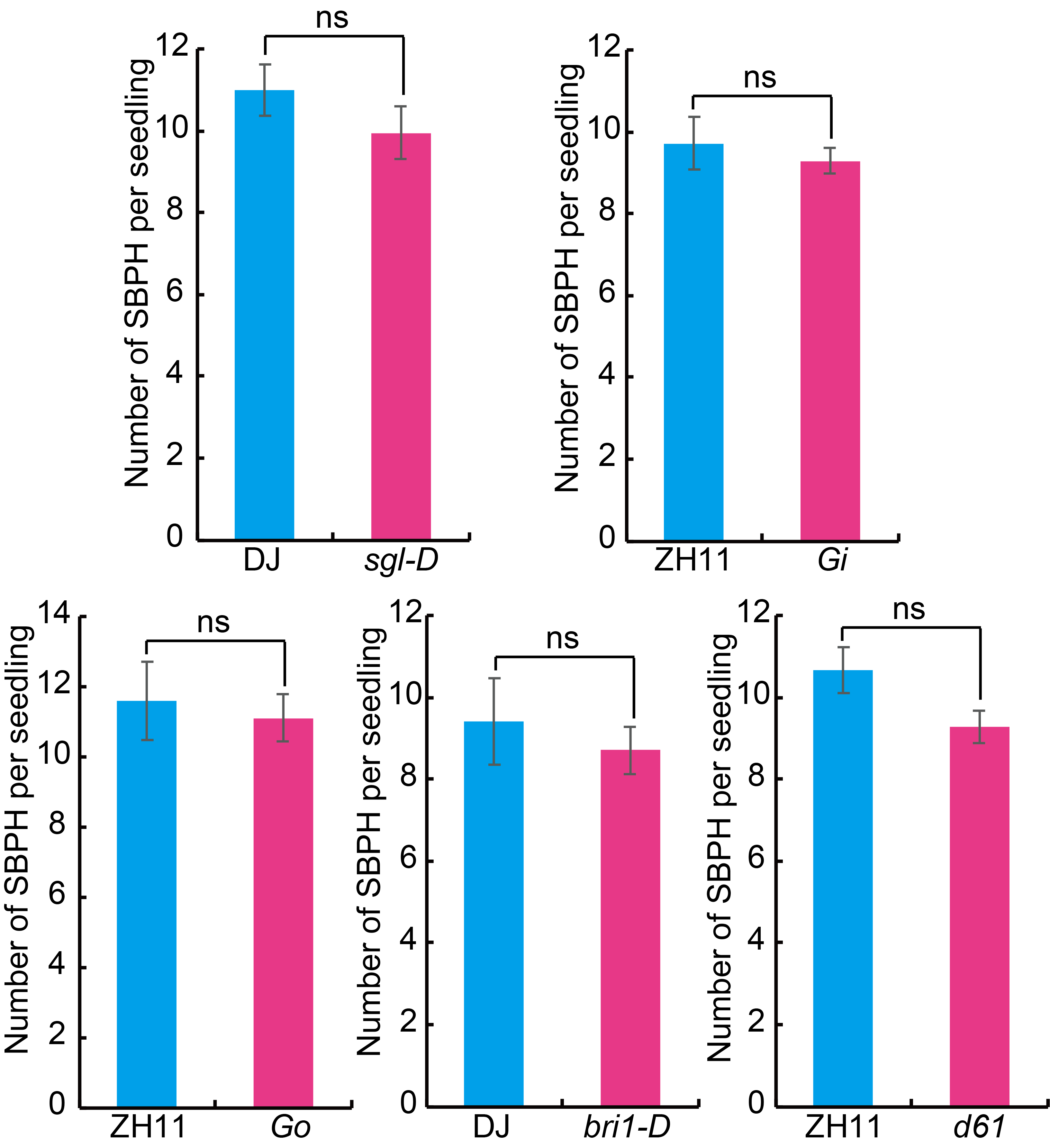

Supplement: S2 Fig — Numbers of SBPH on slg-D, Dongjin (DJ), Gi, Zhonghua11 (ZH11), Go, bri1-D and d61 plants were recorded at 24 h post infestation with SBPH. “ns” indicate no significant difference in comparison with the WT plant (Student’s t-test). (TIF) [file ppat.1008801.s002.tif]

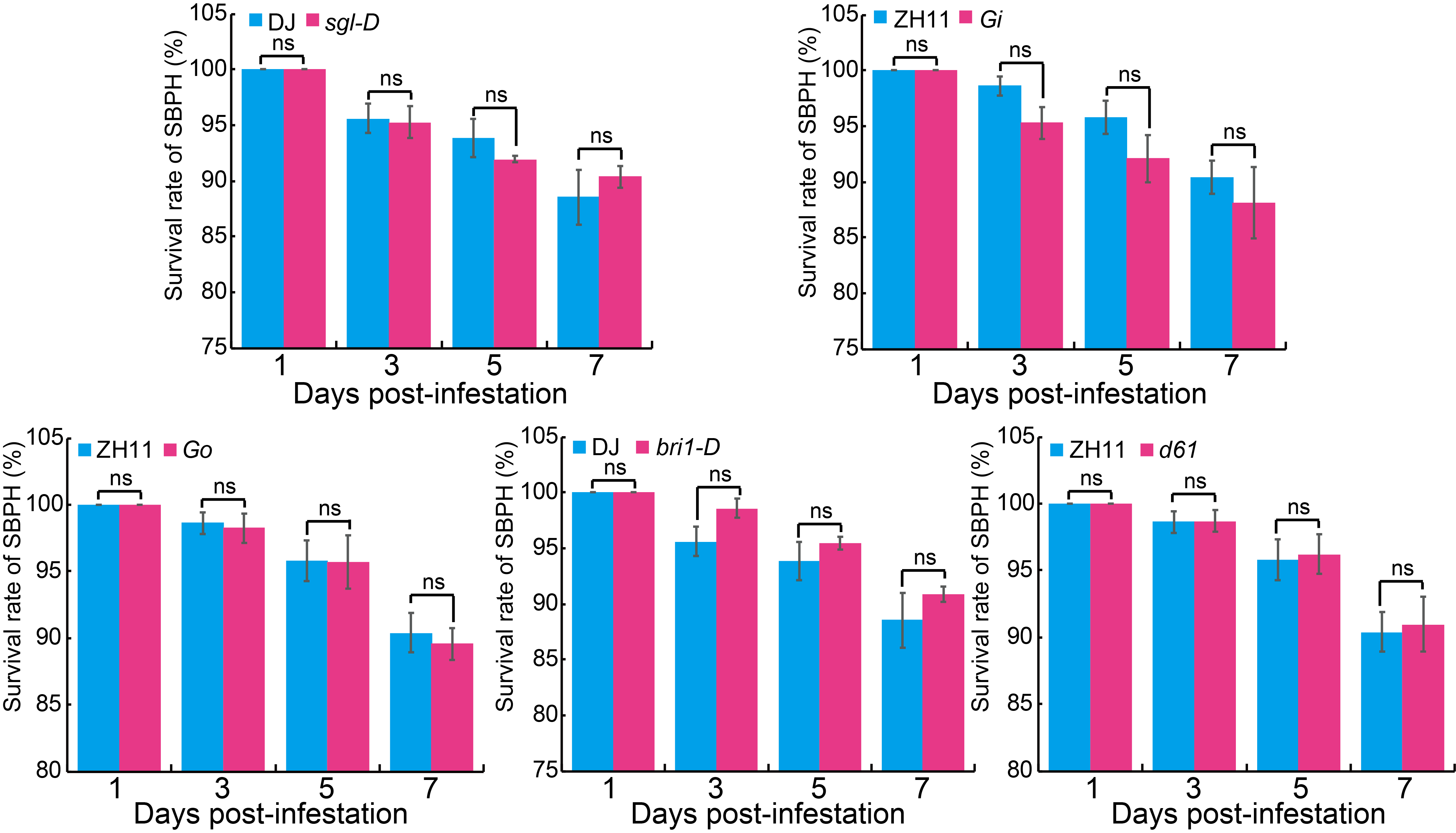

Supplement: S3 Fig — Survival rates of SBPH on slg-D, Dongjin (DJ), Gi, Zhonghua11 (ZH11), Go, bri1-D and d61 plant were recorded on 1, 3, 5 and 7-day post infestation with SBPH. “ns” indicate no significant difference in comparison with the WT plant (Student’s t-test). (TIF) [file ppat.1008801.s003.tif]

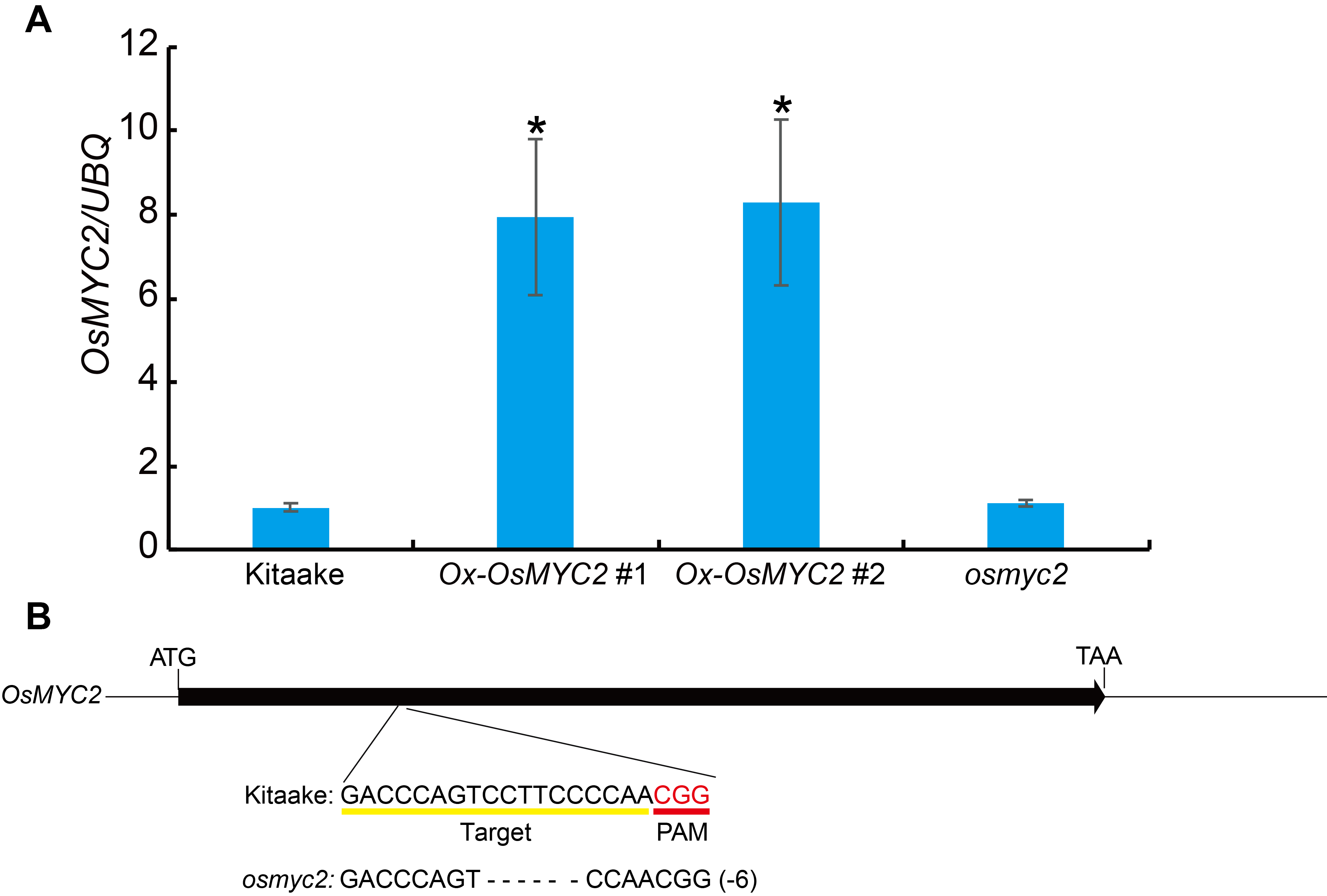

Supplement: S4 Fig — (A) qRT-PCR analysis of the transcript levels of OsMYC2 in Kitaake, OsMYC2 over-expressing (Ox-OsMYC2) and knockout (osmyc2) plants. The expression level of OsMYC2 in Kitaake was set as 1. Ubiquitin (Os03g0234350) was used as internal reference. Data are shown as mean ± SEM (n = 3). * P < 0.05 by Student’s t-test. (B) Diagram of the CRISPR/Cas9 target fragment (in yellow) in the CDS of OsMYC2. The position of the PAM sequence is underlined (in red). The minus signs indicate the base deleted in osmyc2 in comparison with Kitaake. (TIF) [file ppat.1008801.s004.tif]

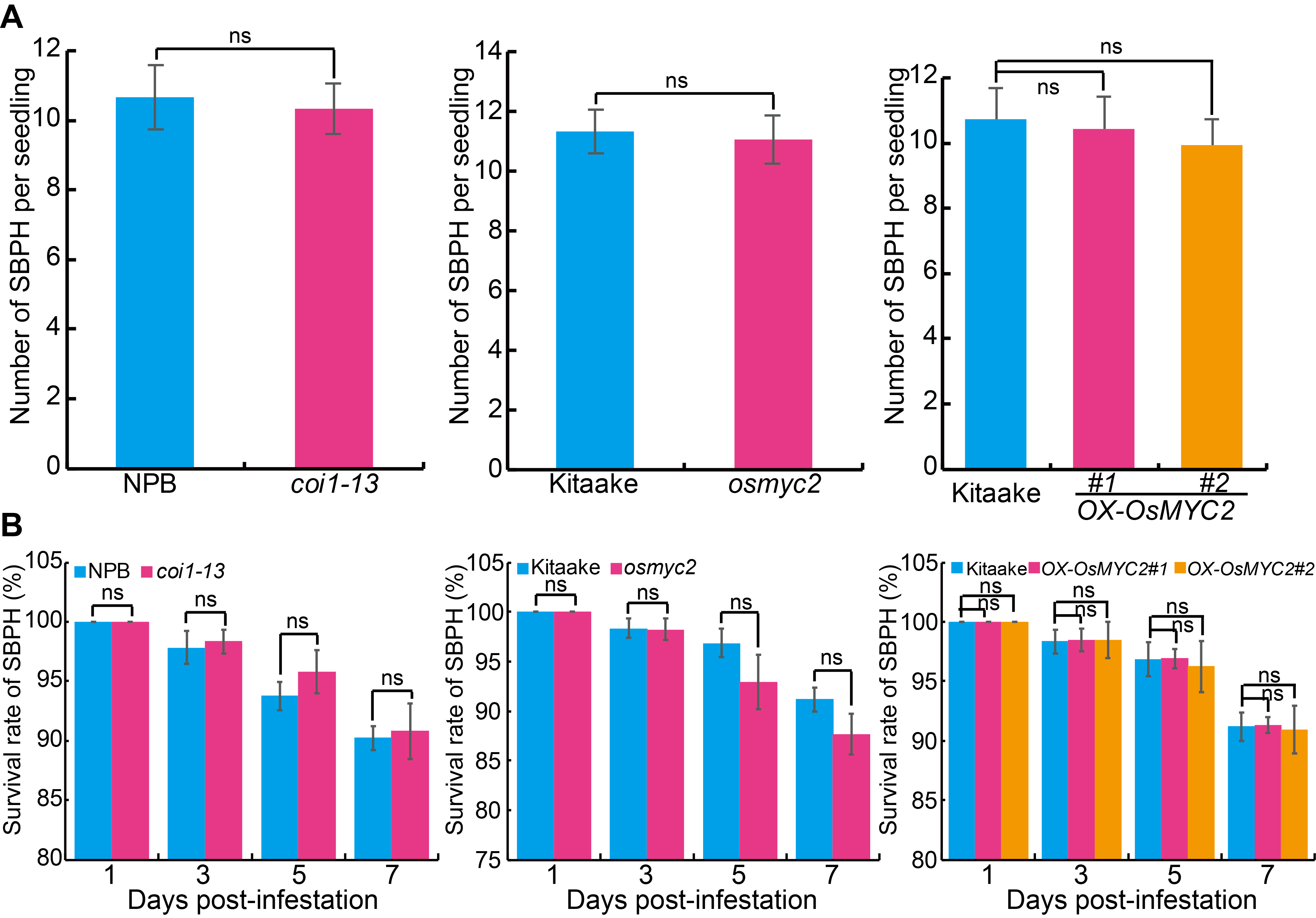

Supplement: S5 Fig — (A) Numbers of SBPH on Nipponbare (NPB), JA co-receptor OsCOI1 RNAi (coi1-13), Kitaake, OsMYC2 knock out (osmyc2) and OsMYC2 overexpressing (Ox-OsMYC2) plants were recorded at 24 h post infestation with SBPH. Data are shown as mean ± SEM (n = 9). (B) Survival rate of SBPH on coi1-13, NPB, osmyc2 and Ox-OsMYC2 plants on 1, 3, 5 and 7-d post infestation with SBPH. Data are shown as mean ± SEM (n = 5). “ns” indicate no significant difference in comparison with the WT plant (Student’s t-test). (TIF) [file ppat.1008801.s005.tif]

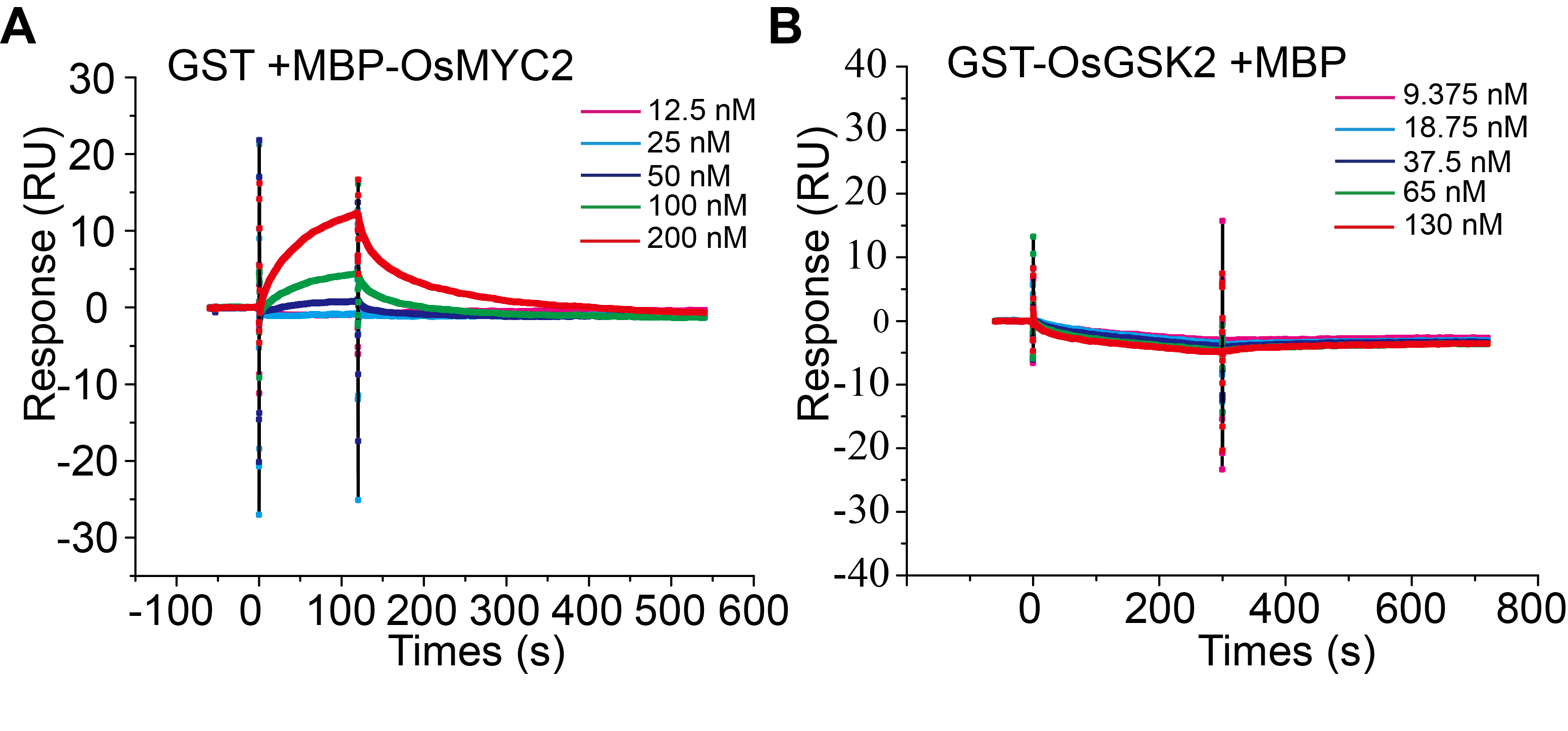

Supplement: S6 Fig — (TIF) [file ppat.1008801.s006.tif]

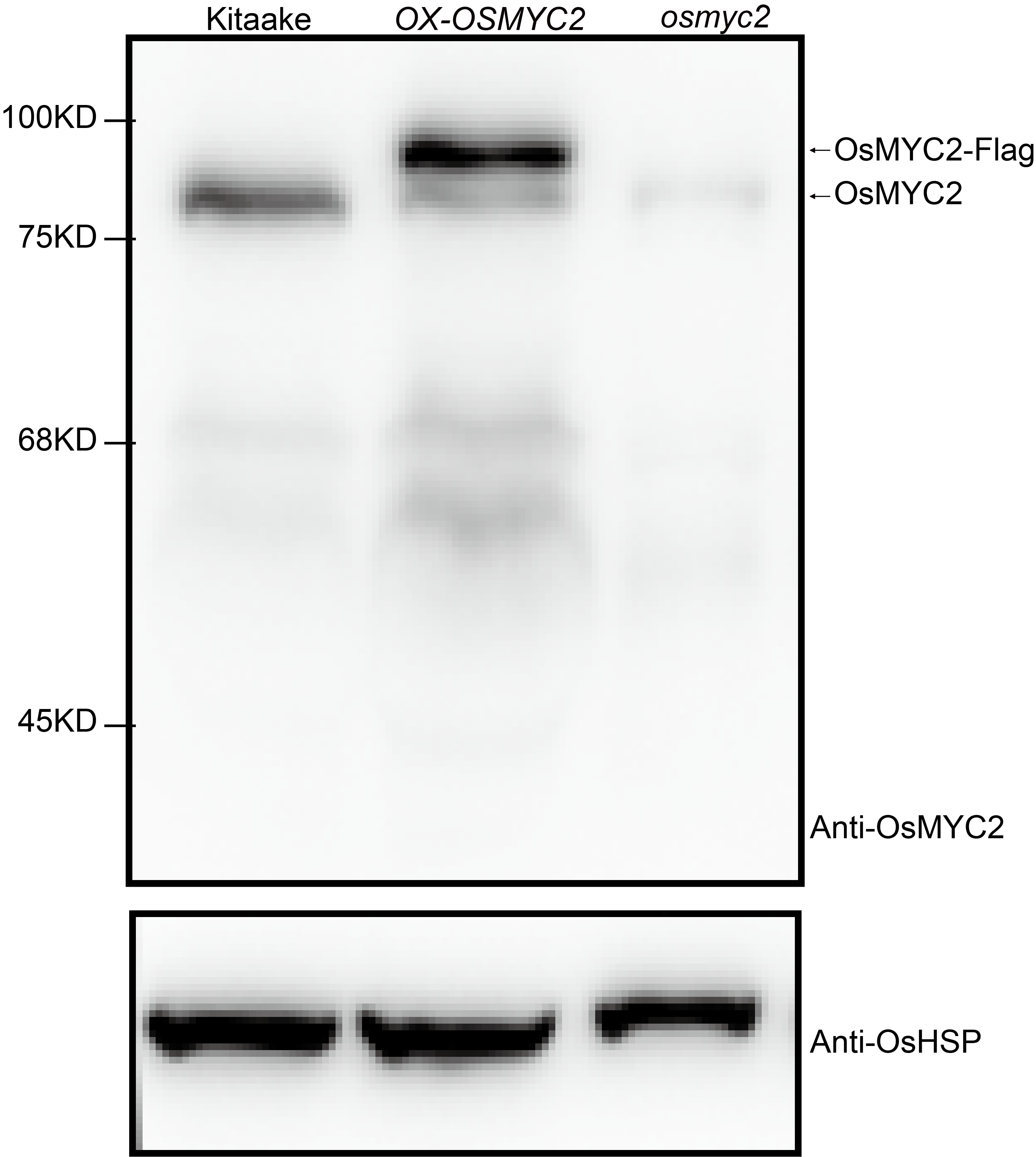

Supplement: S7 Fig — OsHSP was used as an internal reference. (TIF) [file ppat.1008801.s007.tif]

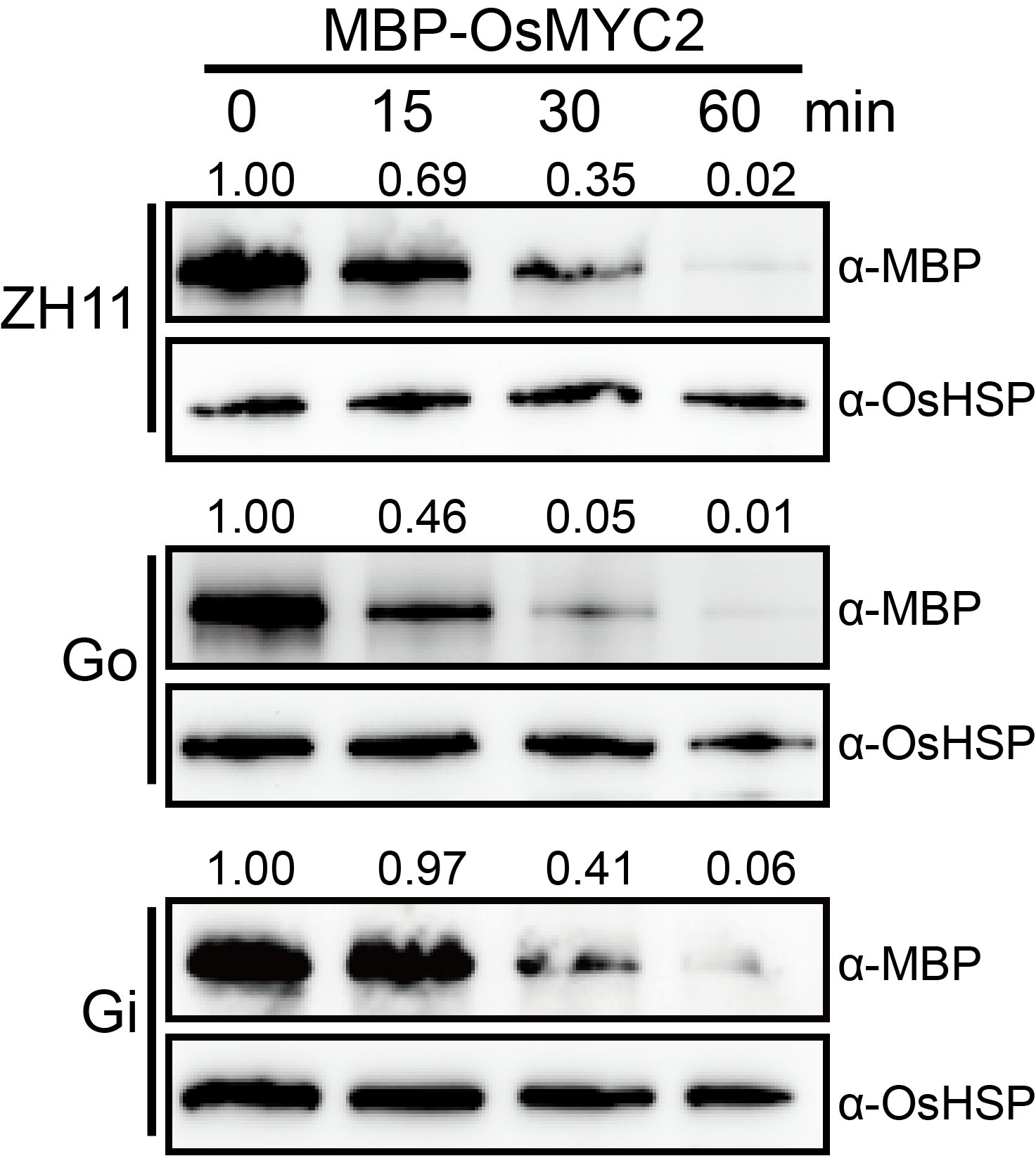

Supplement: S8 Fig — OsHSP was used as loading control. The number on the each panel indicate the protein level of MBP-OsMYC2 relative to its initial value (0 min). (TIF) [file ppat.1008801.s008.tif]

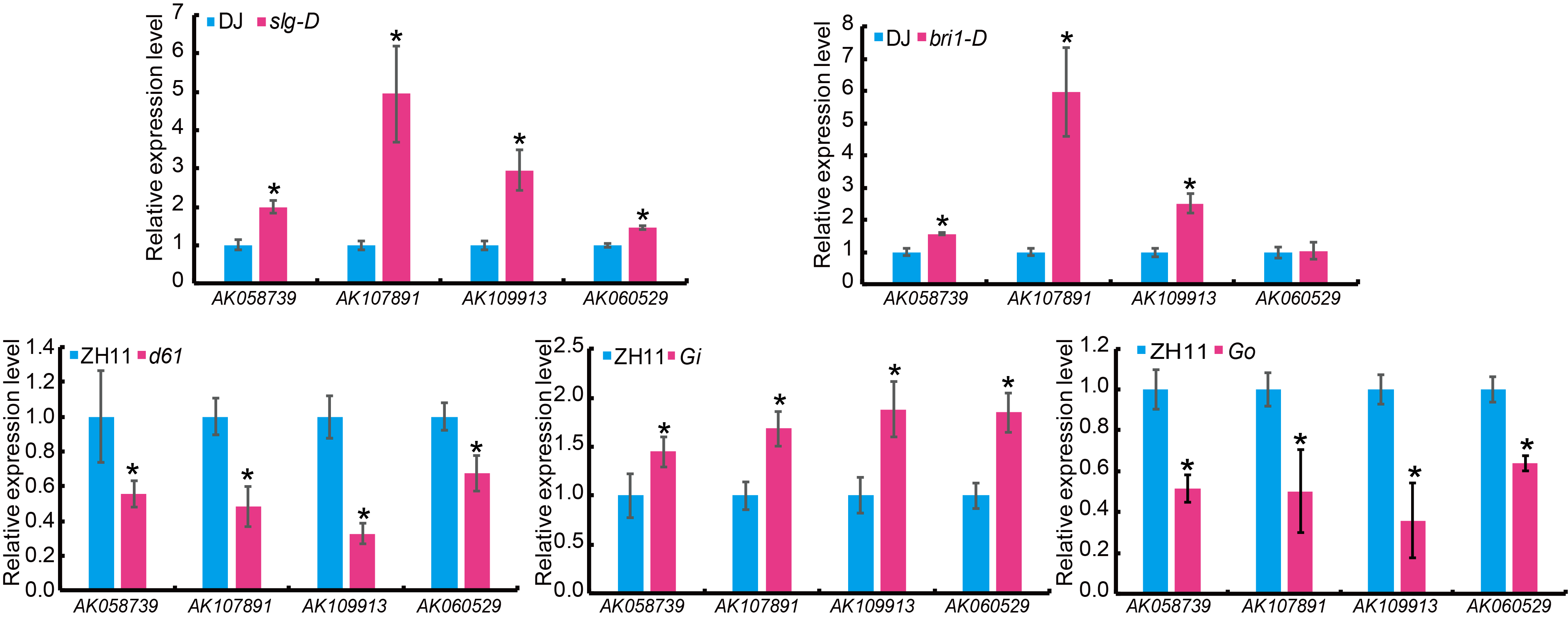

Supplement: S9 Fig — All data are shown as mean ± SEM (n = 3). *P < 0.05 by Student’s t-test. AK058739 (subtilisin/chymotrypsin inhibitor), AK107891 (lipid transfer protein), AK109913 (similar to thaumatin-like protein) and AK060529 (beta-1, 3-glucanase). (TIF) [file ppat.1008801.s009.tif]

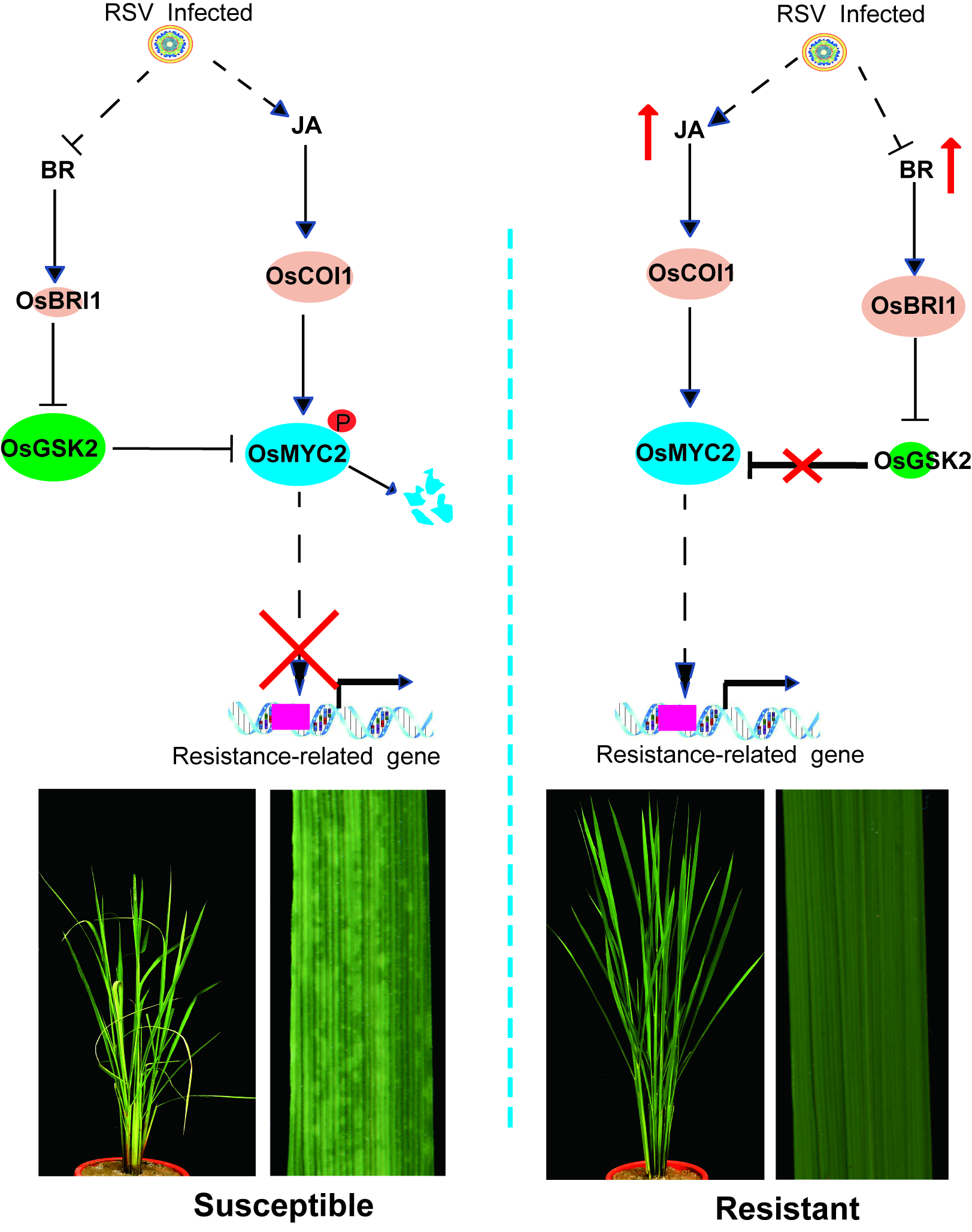

Supplement: S10 Fig — When rice plant is infected with RSV, the biosynthesis of JA is induced, which activates the JA-mediated RSV resistance response. Meanwhile, RSV infection increases the accumulation of OsGSK2 by reducing the level of endogenous BR. OsGSK2 interacts with and phosphorylates OsMYC2, which results in the degradation of OsMYC2 and blocking of the JA signal pathway to benefit viral infection. (TIF) [file ppat.1008801.s010.tif]
