# Supplementary material for: Rice stripe virus suppresses jasmonic acid-mediated resistance by hijacking brassinosteroid signaling pathway in rice
Source: PLoS Pathog. 2020 Aug 31;16(8):e1008801. doi: 10.1371/journal.ppat.1008801 (PMC7485985; doi:10.1371/journal.ppat.1008801)
Supplement: S1 Table — (DOCX) [file ppat.1008801.s011.docx]

**Table S1. Primers used in the study.**

| Primer name | | | Sequence |
| --- | --- | --- | --- |
| **For vector construction** | | | |
| CRISPR-OsMYC2-F | | | AGATGATCCGTGGCAGACCCAGTCCTTCCCCAAGTTTTAGAGCTATGC |
| CRISPR-OsMYC2-R | | | GCATAGCTCTAAAACTTGGGGAAGGACTGGGTCTGCCACGGATCATCT |
| 1300-flag-GSK2-F | | | ACGATGATAAGGGCGGTACCATGGACCAGCCGGCGCCG |
| 1300-flag-GSK2-R | | | AGGCTACGTAGGATCCTTAGCTCCCAGTATTGAA |
| OsMYC2-GFP-F | | | CGGAGCTAGCTCTAGAATGAACCTTTGGACGGACGACA |
| OsMYC2-GFP-R | | | TGCTCACCATGGATCCCCGGGCGGCGGTGCCAGGCT |
| BD-OsGSK2-F | | | CATGGAGGCCGAATTCATGGACCAGCCGGCGCCGGCGCCGGA |
| BD-OsGSK2-R | | | GCAGGTCGACGGATCCTTAGCTCCCAGTATTGAAGAAGTTGTG |
| AD-OsMYC2-F | | | GGAGGCCAGTGAATTCATGAACCTTTGGACGGACGACA |
| AD-OsMYC2-R | | | CGAGCTCGATGGATCCTTACCGGGCGGCGGTGCC |
| nluc-MYC2-F | | | CGGGGGACGAGCTCGGTACCATGAACCTTTGGACGGACGACA |
| nluc-MYC2-R | | | ACGAGATCTGGTCGACCCGGGCGGCGGTGCCAGGCT |
| cluc-GSK2-F | | | ACGCGTCCCGGGGCGGTACCATGGACCAGCCGGCGCCG |
| cluc-GSK2-R | | | AGCTCTGCAGGTCGACTTAGCTCCCAGTATTGAAGAA |
| OsMYC2-pMAL-C2X-F | | | AAGGATTTCAGAATTCATGAACCTTTGGACGGACGACA |
| OsMYC2-pMAL-C2X-R | | | CGACTCTAGAGGATCCTTACCGGGCGGCGGTGCC |
| OsGSK2-pGEX-4T-F | | | TGGATCCCCGGAATTCATGGACCAGCCGGCGCCGGCGCCGGA |
| OsGSK2-pGEX-4T-R | | | GTCGACCCGGGAATTCTTAGCTCCCAGTATTGAAGAAGTTGTG |
| 1300-flag-OsMYC2-F | | | AACACGGGGGACTCTAGAATGAACCTTTGGACGGACGACA |
| 1300-flag-OsMYC2-R | | | CTTGTAGTCCATGTCGACCCGGGCGGCGGTGCCAGGCT |
| **For RT-qPCR** | | | |
| Ubiquitin-F | | GCTCCGTGGCGGTATCAT | |
| Ubiquitin-R | | CGGCAGTTGACAGCCCTAG | |
| RSV-CP-F | | AGTGCTGATCGTATTGACAGA | |
| RSV-CP-R | GATGAAGTACACAACTGGTC | | |
| qD2-F | | | CCTTTTGGTGGTGGGCAGAG |
| qD2-R | | | TGGGGAAGTTGACGATGTGGT |
| qD11-F | | | CAAGGGACAAGCAAGAAGTTTAC |
| qD11-R | | | CGATTTCTATGGGCAGACCTC |
| qOsDWARF4-F | | | TGGGCTCTGAAACAATCTAACCT |
| qOsDWARF4-R | | | CAAGGAAGAAGATGGCGAGG |
| qOsCPD1-F | | | ATGAGACACTCCGTGTGGGT |
| qOsCPD1-R | | | GTTGATCTGCCATCTCCAAG |
| qOsCPD2-F | | | CCGGTTGTGTCCTGGCTATG |
| qOsCPD2-R | | | GATTCTGAAAGCAGCCGAAG |
| qOsBZR1-F | | | AGATGGTTCCTTTCGTGGAC |
| qOsBZR1-R | | | AGAATGAAATCGCCCAAATC |
| qOsBZR2-F | | | AACAACGAGGTCCTCAAGGC |
| qOsBZR2-R | | | GGAACACGGGCTTGGTGATA |
| qOsGSK2-F | | | CAGTTATTTAGGGGGCTTGCGT |
| qOsGSK2-R | | | CTCGGTAGTAGCGTGAGCATA |
| qOsBRI1-F | | | GCAAGGGTATCTGATTTCGGT |
| qOsBRI1-R | | | CAAGAGTGGACACGCTAAGGT |
| qOsMYC2-F | | | AAGCTCAACCAGCGCTTCTA |
| qOsMYC2-R | | | CGGAGCTCGTTGATGTAGGA |
| qOsLOX1-F | | | GTACGCTGGGTTCACAGCTC |
| qOsLOX1-R | | | TCAGATGGATGTGCTGTTGG |
| qOsLOX5-F | | | CTGATGAGGAGTTTGCACGA |
| qOsLOX5-R | | | TCGTCCTTCAGGAGCAGAAT |
| qAK058739-F | | | GTTTGATCGTGTCCGGAGGT |
| qAK058739-R | | | ATCACCTTCTTCGCCTCCTC |
| qAK107891-F | | | CGACCTACTTCACCAACTGC |
| qAK107891-R | | | GCACCTGAGGTTTCTCATGC |
| qAK109913-F | | | CTCGCAGGTGTTCAAGTCG |
| qAK109913-R | | | GCAGAAGGTGAGGGTGTAGT |
| qAK060529-F | | | GTGTACCCGTACTTCGCCTA |
| qAK060529-R | | | GCGTCGAACATGTTGGTGTA |
|  | | |  |
